# Maladaptive functional changes in alveolar fibroblasts due to perinatal hyperoxia impair epithelial differentiation

**DOI:** 10.1172/jci.insight.152404

**Published:** 2022-03-08

**Authors:** Matthew R. Riccetti, Mereena George Ushakumary, Marion Waltamath, Jenna Green, John Snowball, Sydney E. Dautel, Mehari Endale, Bonny Lami, Jason Woods, Shawn K. Ahlfeld, Anne-Karina T. Perl

**Affiliations:** 1 The Perinatal Institute and Section of Neonatology, Perinatal and Pulmonary Biology, and; 2 Molecular and Developmental Biology Graduate Program, Cincinnati Children’s Hospital Medical Center, Cincinnati, Ohio, USA.; 3 Department of Pediatrics, University of Cincinnati College of Medicine, Cincinnati, Ohio, USA.; 4 Center for Pulmonary Imaging Research, Division of Pulmonary Medicine & Department of Radiology, Cincinnati Children’s Hospital, Cincinnati, Ohio, USA.

**Keywords:** Development, Pulmonology, Fibrosis, Growth factors, Mouse models

## Abstract

Infants born prematurely worldwide have up to a 50% chance of developing bronchopulmonary dysplasia (BPD), a clinical morbidity characterized by dysregulated lung alveolarization and microvascular development. It is known that PDGFR alpha–positive (PDGFRA^+^) fibroblasts are critical for alveolarization and that PDGFRA^+^ fibroblasts are reduced in BPD. A better understanding of fibroblast heterogeneity and functional activation status during pathogenesis is required to develop mesenchymal population–targeted therapies for BPD. In this study, we utilized a neonatal hyperoxia mouse model (90% O_2_ postnatal days 0–7, PN0–PN7) and performed studies on sorted PDGFRA^+^ cells during injury and room air recovery. After hyperoxia injury, PDGFRA^+^ matrix and myofibroblasts decreased and PDGFRA^+^ lipofibroblasts increased by transcriptional signature and population size. PDGFRA^+^ matrix and myofibroblasts recovered during repair (PN10). After 7 days of in vivo hyperoxia, PDGFRA^+^ sorted fibroblasts had reduced contractility in vitro, reflecting loss of myofibroblast commitment. Organoids made with PN7 PDGFRA^+^ fibroblasts from hyperoxia in mice exhibited reduced alveolar type 1 cell differentiation, suggesting reduced alveolar niche-supporting PDGFRA^+^ matrix fibroblast function. Pathway analysis predicted reduced WNT signaling in hyperoxia fibroblasts. In alveolar organoids from hyperoxia-exposed fibroblasts, WNT activation by CHIR increased the size and number of alveolar organoids and enhanced alveolar type 2 cell differentiation.

## Graphical abstract

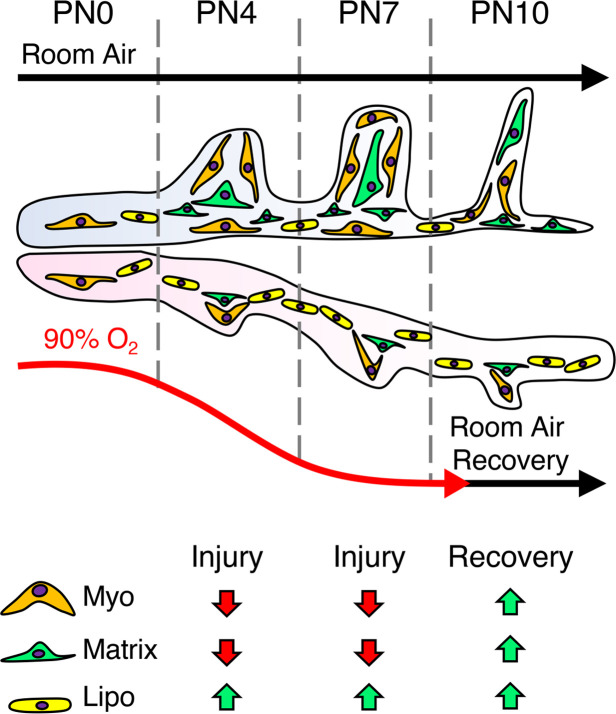

## Introduction

Humans are born during the alveolar phase of lung development, when secondary crests form and newly forming alveolar walls extend into the airway lumen to increase surface area for gas exchange (1, 2). Premature infants born during the saccular phase of development require life-saving oxygen supplementation and ventilation, which can inhibit alveologenesis, damage the existing lung, and lead to the formation of “new” bronchopulmonary dysplasia (BPD) (3–5). “New” BPD pathology is characterized by reduced alveolar surface area, expanded airspace, thinned septal walls, inflammation, and aberrant differentiation of the epithelium, the endothelium, and mesenchymal populations (4, 6, 7). It is known that genetic disruption of PDGF alpha (PDGFA) signaling in the distal pulmonary mesenchyme in mice causes permanent alveolar simplification in a manner similar to BPD, suggesting that disruption of mesenchymal development is involved in BPD pathogenesis (8). A better understanding of the function and regulation of mesenchymal subpopulations during alveolarization may lead to targeted therapies for BPD.

Within the mesenchyme, PDGFRA^+^ fibroblasts are a heterogenous cell population that reside in the distal lung and are critical for proper secondary septation during alveologenesis (9–13). PDGFRA^+^ fibroblasts can be separated into 3 functionally distinct fibroblast populations: secondary crest myofibroblasts, lipofibroblasts, and matrix fibroblasts (9, 14–21). Secondary crest myofibroblasts form and contract the alveolar entry ring during alveolarization and express *Cd29*, *Fgf18*, *Acta2*, *Gli1*, and *Pdgfra* (2, 17, 19, 21–26). Myofibroblasts contract the ECM and are absolutely required for alveolarization but are known to aberrantly populate the lung in patients with BPD and BPD animal models (27–30). Lipofibroblasts provide neutral lipids and paracrine ligands to alveolar type 2 epithelial cells (AT2 cells) and express *Pdgfra*, *Tcf21*, *Plin2*, and *Fgf10* (17, 31–34). Lipofibroblasts are thought to contribute to myofibroblasts during BPD progression and are reduced in the lungs of patients with BPD (30, 35, 36). Matrix fibroblasts secrete ECM components and ECM-modifying proteins and have been associated with high expression of *Pdgfra* and *Cd34* (16). Matrix fibroblasts support alveolar type 1 cell (AT1 cell) differentiation via paracrine signaling and are highly responsive to PDGFA, but their role in BPD pathogenesis is currently unknown (14, 16, 19, 37). Previous studies demonstrate that PDGFRA^+^ fibroblasts are reduced in patients with BPD and BPD animal model lungs (38–40), and genetic ablation of PDGFRA^+^ fibroblasts during alveolarization causes persistent alveolar simplification (12). It remains unknown what dynamic changes occur in the heterogenous mix of PDGFRA^+^ fibroblasts (matrix fibroblasts, myofibroblasts, and lipofibroblasts) during neonatal hyperoxia exposure and room air (RA) recovery and how altered function of PDGFRA^+^ fibroblasts or changed contribution of PDGFRA^+^ fibroblast subpopulations result in impaired alveolar formation and alveolar epithelial differentiation.

Unlike full-term human babies, mice are born during the saccular stage of lung development; thus, hyperoxia exposure of neonatal mice is a model of hyperoxia exposure of premature human babies and allows us to study some aspects resulting in the formation of BPD (6, 41). Exposure of neonatal mice to hyperoxia (90% O_2_) from birth (P0) inhibits secondary septation and microvascular development and results in persistent diminished alveolar surface area, abnormalities in alveolar matrix structure, and reduced alveolar vessel density, replicating a subset of the morphological and functional pathology of human BPD (42–47). To perturb alveolar septal and microvascular development with a developmentally and clinically relevant model of neonatal lung injury, we exposed neonatal mice to hyperoxia between postnatal days 0 and 7 (PN0 and PN7) and harvested the lungs at PN4 (saccular-to-alveolar transition), PN7 (peak alveolarization), and PN10 (ECM remodeling during alveolarization). We isolated fresh PDGFRA^+^ fibroblasts by magnetic-activated cell sorting and performed bulk RNA-Seq, flow cytometry, and functional assays during early hyperoxia injury (PN0, PN7) and hyperoxia recovery (PN10) to dissect changes in cell activation state and function. In this study, we (i) define dynamic changes in gene expression and function in PDGFRA^+^ fibroblasts during hyperoxia injury and recovery, (ii) provide evidence that loss of AT1 cell differentiation in hyperoxia is in part due to loss of PDGFRA^+^ matrix/myofibroblasts, and (iii) show that activation of canonical WNT signaling or PDGFRA signaling in organoid culture with primary hyperoxia fibroblasts restores alveolar organoid formation and AT2 and AT1 cell differentiation.

## Results

### Transcriptional profiling reveals dynamic changes in PDGFRA^+^ fibroblast proliferation and functional differentiation during and after neonatal hyperoxia exposure.

**Figure 1:**
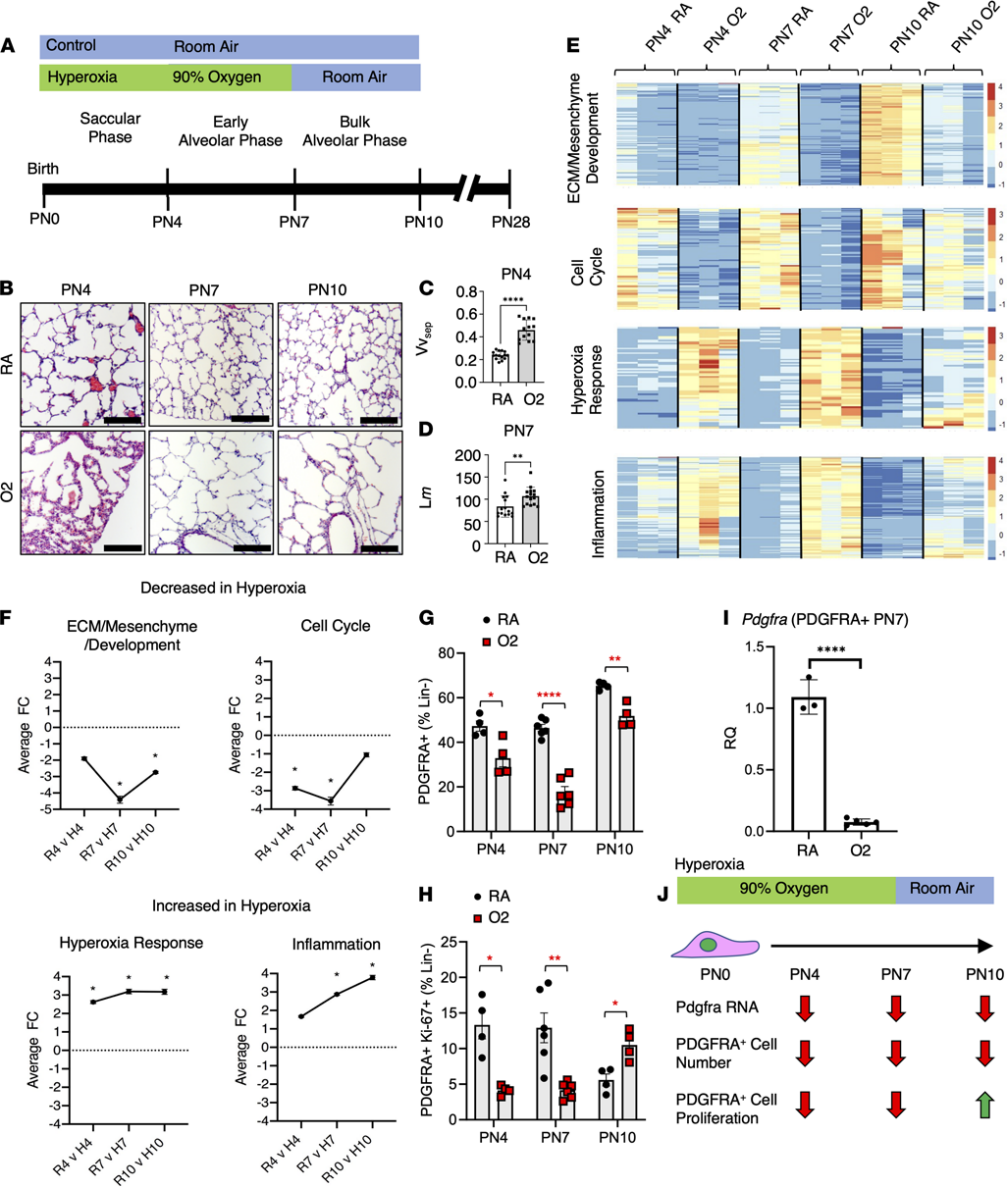
Exposure to hyperoxia PN0–PN7 results in increased inflammatory and hypoxic response and decreased cell cycle and ECM development gene expression in isolated PDGFRA^+^ fibroblasts. (**A**) Timeline and murine hyperoxia exposure schematic used. (**B**) H&E of PN4, PN7, and PN10 RA and hyperoxia (O2) lungs. Scale bars = 250 μm. (**C**) Vv_sep_ (volume density of alveolar septa) of PN4 RA and O2 lungs. (**D**) L*m* (mean linear intercept of airspaces) of PN7 RA and O2 lungs. In **C** and **D**, *n* = 3. Two-tailed Student’s *t* test was used, **P < 0.01; ****P < 0.0001. Error bars show mean ± SD. (**E**) Gene enrichment analysis performed using ToppGene’s ToppFun, functional enrichments within each profile identified, all profiles compared with each other using Toppcluster. Heatmap of the resulting list, *z* score normalized, generated using Partek Genomics Suite (85). (**F**) Average fold change of associated GO terms for each time point in O2 compared with RA. (**G**) Flow cytometry on PDGFRA^+^ fibroblasts performed at PN4, PN7, and PN10 and gated as CD45^–^CD326^–^CD31^–^ (Lineage-negative fibroblasts, “Lin^–^”) and CD140^+^ (PDGFRA^+^). PDGFRA^+^ over Lin^–^ reveals reduction in total PDGFRA^+^ fibroblasts in O_2_. (**H**) PDGFRA^+^Ki-67^+^ compared with total Lin^–^ fibroblasts reveals reduced proliferation in O2 until PN10, when it was increased compared with RA. In **G** and **H**, *n* = 4–6 RA and O2 mice were used. Two-tailed Student’s *t* test was used, *P < 0.05; **P < 0.01; ****P < 0.0001. Error bars show mean ± SEM. (**I**) MACS-isolated PDGFRA^+^ fibroblasts show reduced *Pdgfra* expression by RT-qPCR. *n* = 3–6 RA and O_2_ mice. Two-tailed Student’s *t* test was used, ****P < 0.0001. Error bars show mean ± SD. (**J**) Schematic showing loss of PDGFRA cell number and proliferation during hyperoxia early injury and recovery.

Neonatal pups were exposed to 90% O_2_ hyperoxia (O_2_) or RA starting at PN0 and harvested during O_2_ exposure (PN4, PN7) or after recovery (PN10) (Figure 1A). Morphologic evaluation at PN4, PN7, and PN10 supported previous findings of hyperoxia-induced lung damage (Figure 1B). At PN4, hyperoxia-exposed lungs showed increased volume density of alveolar septa and reduced mean linear intercept of the airspaces, indicating a delay in septal wall thinning (Figure 1C and Supplemental Figure 1C; supplemental material available online with this article; https://doi.org/10.1172/jci.insight.152404DS1). At PN7, hyperoxia-exposed lungs had increased mean liner intercept, indicating alveolar simplification due to impaired septation (Figure 1D). The alveolar simplification remained during RA recovery at PN10 and PN28 (Supplemental Figure 1, A–E). Hyperoxia injury impaired AT2 to AT1 differentiation in vivo (48). IHC on hyperoxia-exposed lungs from PN7 for homeodomain-only protein homeobox (HOPX), AGER (AT1), and SPC (AT2) revealed a reduction in AT1 cells at PN7. While gene expression of *Hopx* and *Pdgfa* in PN7 magnetic-activated cell sorting–isolated (MACS-isolated) hyperoxia CD326^+^ epithelium transcripts were increased in quantitative reverse transcription PCR (RT-qPCR), suggesting that the changes are at a protein rather than an RNA level (Supplemental Figure 1, F–H).

To determine dynamic changes in gene expression within the PDGFRA^+^ population, we performed bulk RNA-Seq on MACS PDGFRA^+^ cells from RA and hyperoxia-exposed PN4, PN7, and PN10 murine lungs. Gene Ontology (GO) analysis on differentially expressed genes (fold change > 2 and *P* < 0.01) was performed to identify trends in cell proliferation and function (Figure 1E and Supplemental Figure 2A). “Cell cycle” genes were significantly downregulated in hyperoxia PN4 and PN7, suggesting that hyperoxia reduces proliferation of PDGFRA^+^ fibroblasts (Figure 1F). During recovery, cell cycle genes were upregulated, nearly returning to the levels of RA-exposed fibroblasts, indicating a compensatory proliferation response (Figure 1F). “Hyperoxia response” and “inflammation” genes increased in hyperoxia compared with RA exposure at PN4 and PN7 and reduced expression during recovery at PN10 (Figure 1F), indicating a recovering response. However, “ECM/mesenchyme/development” production genes were suppressed in hyperoxia PDGFRA^+^ fibroblasts at all time points, with highest significance at PN7 and PN10 (Figure 1, E and F), indicating an irreversible loss of matrix modulating function. These gene expression studies suggest that during oxygen injury and dysregulated alveolarization, PDGFRA^+^ fibroblasts are unable to proliferate, undergo cellular stress response, and fail to perform their normal functions of ECM production and maintenance.

### Reduced proliferation and total number of PDGFRA^+^ fibroblasts in hyperoxia at PN4, PN7, and PN10.

To validate predicted changes in the cell cycle of PDGFRA^+^ fibroblasts, we performed flow cytometry on cells isolated from PN4, PN7, and PN10 RA and hyperoxia lungs. Single-cell lung digests were labeled with fluorescent antibodies and subjected to flow cytometry. Fibroblasts were identified by negative selection of CD45^–^/hematopoietic, CD326^–^/epithelial, and CD31^–^/endothelial (Lin^–^). All Lin^–^ fibroblasts were gated for PDGFRA^+^ and Ki-67 (proliferation) (Supplemental Figure 2B). Total Lin^–^ fibroblasts were significantly reduced in hyperoxia at PN7 (Supplemental Figure 2C), replicating observations that the development of the mesenchyme is impaired in BPD (1, 45, 49). Lin^–^ fibroblasts can be divided into PDGFRA– and PDGFRA^+^ fibroblasts, which we hypothesized would have a different proliferative and activation response to hyperoxia (16, 17). In Lin^–^ fibroblasts the number of PDGFRA^–^ fibroblasts were increased and PDGFRA^+^ fibroblasts were reduced at all time points in hyperoxia (Figure 1G and Supplemental Figure 2D). Proliferating Ki67^+^PDGFRA^+^ cells were significantly reduced in hyperoxia lungs at PN4 and PN7 but were significantly increased at PN10 during recovery (Figure 1H and Supplemental Figure 2E). Even with increased proliferation, the number of hyperoxia PDGFRA^+^ fibroblasts did not recover to RA levels by PN10 (Figure 1G). Proliferation followed a similar trend in the PDGFRA^–^ population (Supplemental Figure 2F). These data demonstrate that proliferation is blunted in all fibroblasts during hyperoxia and increases during recovery. But these data do not explain the reduced number of PDGFRA^+^ cells in hyperoxia. We therefore assessed *Pdgfra* gene expression on MACS-isolated PDGFRA^+^ fibroblasts, which was significantly downregulated in hyperoxia fibroblasts at PN7 (Figure 1I). These data demonstrate that PDGFRA is expressed in fewer fibroblasts and at lower levels, suggesting a reduced commitment to the pool of PDGFRA^+^ fibroblasts. The proliferation and gene expression level changes in the PDGFRA^+^ fibroblast population during hyperoxia injury are summarized in Figure 1J.

### Hyperoxia-mediated reduction of PDGFRA^+^ myofibroblast differentiation.

**Figure 2:**
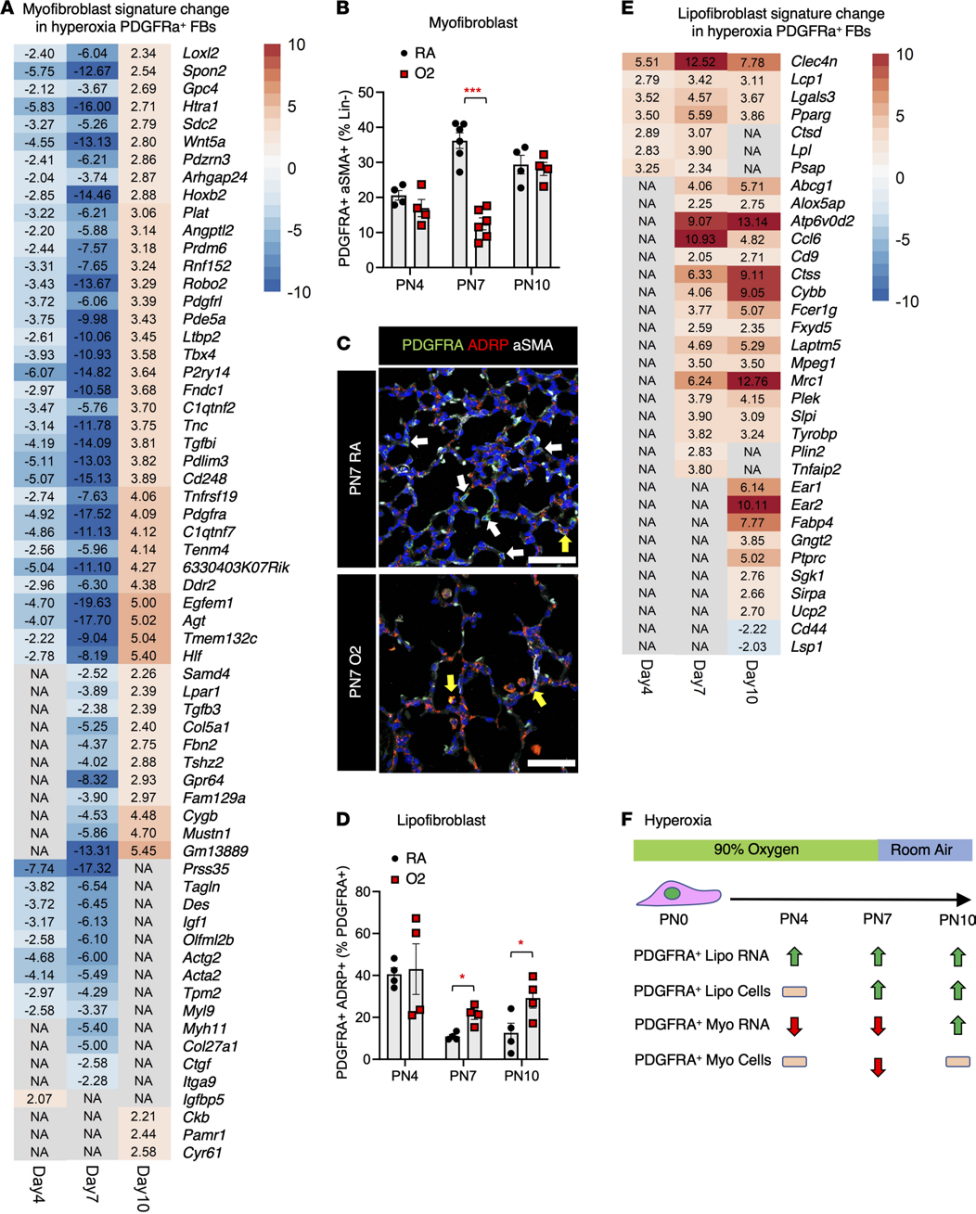
Loss of PDGFRA^+^ myofibroblasts and gain of PDGFRA^+^ lipofibroblasts during alveolarization in hyperoxia-exposed lungs. (**A**) Signature genes for myofibroblasts were determined by downloading and comparing PN7 and PN10 markers genes for respective cell types from LGEA (51). Significant fold change (fold change > 2, binomTest *P* < 0.01, and reads per kilobase of transcript per million mapped reads > 1 in 2 of the 3 replicates in at least 1 condition being compared) displayed in a heatmap made in pheatmap (86). (**B**) Flow cytometry of PDGFRA^+^α-SMA^+^ fibroblasts compared with total Lin^–^ fibroblasts reveals significant reduction of PDGFRA^+^ myofibroblasts at PN7 in O2. (**C**) PN7 RA and O2 lungs immunostained for PDGFRA, ADRP, and α-SMA. Yellow arrows point to PDGFRA^+^ADRP^+^ cells, which are low in RA and enriched in O2, while white arrows point to PDGFRA^+^ADRP^–^ cells; scale bars = 50 μm. (**D**) Flow cytometry on PDGFRA^+^ADRP^+^ compared with total PDGFRA^+^ reveals that, among the PDGFRA^+^ population, there is a selection for lipofibroblasts at PN7 and PN10. In **B** and **D**, *n* = 4–6; control (RA) and experimental (O2) mice were used. A 2-tailed Student’s *t* test was used, *P < 0.05; ***P < 0.001. Error bars show mean ± SEM. (**E**) Top 50 lipofibroblast signature genes were obtained from a recently published mouse lung scRNA-Seq study, with heatmap of significant fold change created using pheatmap (86). (**F**) Schematic that shows dynamic changes in PDGFRA^+^ myo- and lipofibroblast RNA expression and cell populations.

It is well described that thickened bands of myofibroblasts are present in the distal lung of patients with BPD after hyperoxia exposure, but how these myofibroblast bands develop during hyperoxia is poorly understood (36, 50). We generated a “developmental” myofibroblast signature gene list by extracting shared gene expression from the single-cell RNA-Seq data set of myofibroblasts from PN7 and PN10 available on lung gene expression analysis/LungMAP (LGEA/LungMAP) (51–53). Using this myofibroblast signature, we compared gene expression between RA and hyperoxia fibroblasts at PN4, PN7, and PN10. This developmental myofibroblast signature was significantly downregulated in hyperoxia fibroblasts at PN4 and PN7 but upregulated at PN10 (Figure 2A). The number of myofibroblast genes decreased during hyperoxia from PN4 to PN7, indicating progressive loss of myofibroblast commitment among the PDGFRA^+^ fibroblasts during hyperoxia injury. During recovery from hyperoxia (PN10), while many myofibroblast signature genes became upregulated, *Acta2* expression remained unchanged, suggesting that the entire myofibroblast signature has multiple upstream regulators that are differentially affected by hyperoxia.

To determine how hyperoxia changed PDGFRA^+^ fibroblast differentiation to myofibroblasts, we stained for the myofibroblast marker alpha-smooth muscle actin (α-SMA) and performed flow cytometry (Supplemental Figure 3A) (11, 16, 20, 54). There was a significant reduction of hyperoxia PDGFRA^+^α-SMA^+^ myofibroblasts over Lin^–^ fibroblasts at PN7 (Figure 2B). We identified by IHC that the loss of PDGFRA^+^α-SMA^+^ myofibroblasts was most apparent in regions of alveolar simplification in hyperoxia at PN7 (Figure 2C) but also visible at PN4 and PN10 (Supplemental Figure 3B). We previously demonstrated that distal lung myofibroblasts decrease during early lung injury (P0–P7), contrary to the massive increase during prolonged injury (55, 56). However, significantly more PDGFRA^–^ fibroblasts expressed α-SMA at all 3 time points after hyperoxia (Supplemental Figure 3C). These data suggest that hyperoxia specifically reduced myofibroblast differentiation in PDGFRA^+^ fibroblasts (Figure 2F).

### Hyperoxia induces lipofibroblast differentiation during alveolarization.

Current literature reports a reduction of total lipofibroblasts in hyperoxia, but this does not account for changes in PDGFRA-positive or -negative fibroblasts (34, 57–59). We used the lipofibroblast marker adipocyte differentiation-related protein (ADRP) to assess lipofibroblast differentiation in PDGFRA^+^ fibroblasts. Hyperoxia-exposed PDGFRA^+^ lipofibroblasts (PDGFRA^+^ADRP^+^) increased in total numbers compared with RA-exposed PDGFRA^+^ lipofibroblasts at PN7 and PN10 (Figure 2D). We confirmed an increase in lipofibroblast gene expression at all 3 time points in hyperoxia PDGFRA^+^ cells, with 7 lipofibroblast genes up at PN4, 24 up at PN7, and 27 up at PN10 (Figure 2E). *Pparg*, a nuclear receptor known to induce lipofibroblast fate in PDGFRA^+^ fibroblasts (33, 60), was upregulated at PN4. However, *Plin2* gene expression and ADRP protein expression were observed at PN7 (Figure 2, C and E). These data suggest that in hyperoxia, PN4 PDGFRA^+^ fibroblasts are committed to lipofibroblast fate by PN4 and fully differentiate into lipofibroblasts by PN7 (Figure 2F).

### Hyperoxia suppresses PDGFRA^+^ matrix fibroblast commitment during alveolarization.

**Figure 3:**
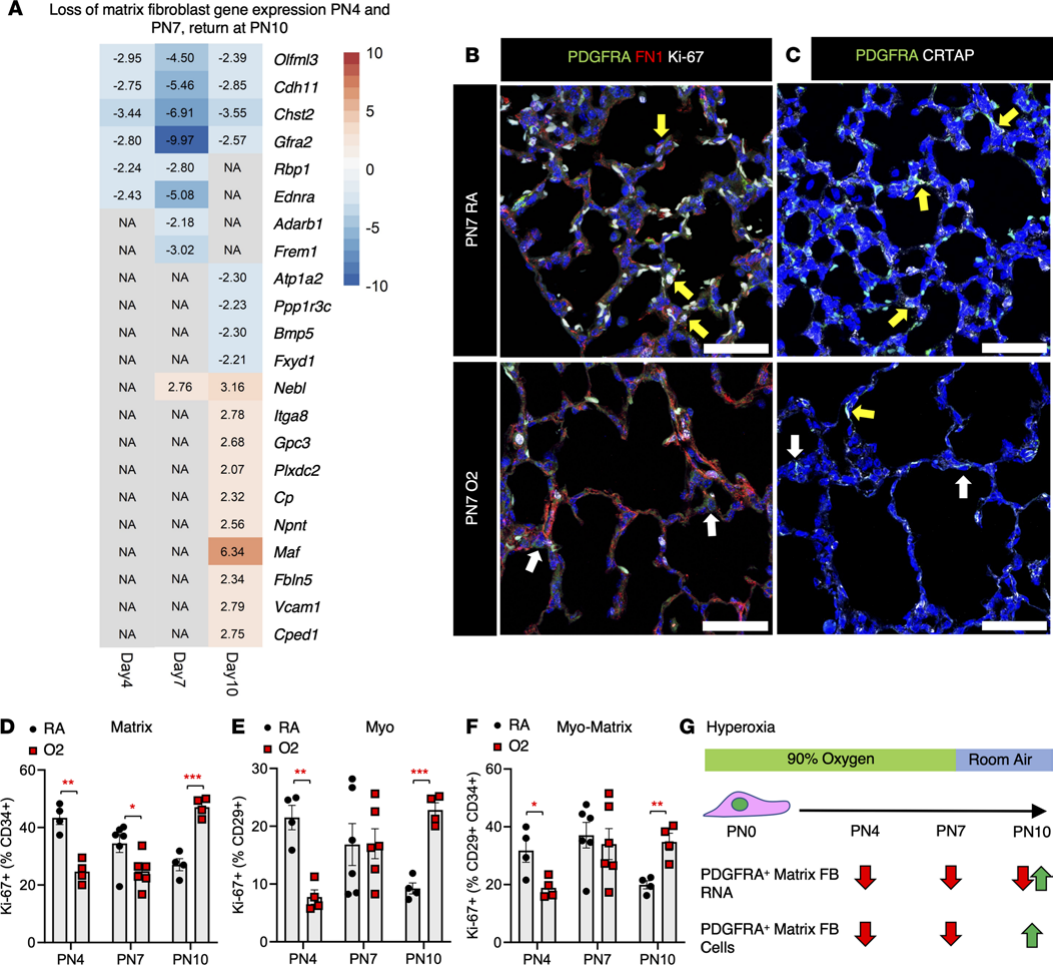
Loss of PDGFRA^+^ myo/matrix fibroblasts in hyperoxia. (**A**) Heatmap made in the pheatmap program of significantly changed matrix-fibroblast signature genes, identified using LGEA (https://research.cchmc.org/pbge/lunggens/mainportal.html) (51, 86) (**B**) PN7 RA and O2 immunofluorescence for PDGFRA (green), FN1 (red), and Ki-67 (white). Yellow arrows point to proliferating (nuclear Ki-67) PDGFRA^+^FN1^+^ matrix fibroblasts (FBs), which are largely absent from O2 lungs. White arrows point to nonproliferating PDGFRA^+^FN1^–^ FBs. (**C**) PN7 RA and O2 immunofluorescence showing reduced matrix fibroblast marker CRTAP (white) in PDGFRA^+^ fibroblasts (green) in hyperoxia. Yellow arrows point to PDGFRA^+^CRTAP^+^ matrix fibroblasts, white arrows point to PDGFRA^+^CRTAP^–^ FBs. In **B** and **C**, scale bars = 50 μm. (**D**–**F**) Flow cytometry. PDGFRA^+^ fibroblasts were gated using CD29 on the *x* axis and CD34 on the *y* axis (16, 17). (**D**) CD34^+^Ki-67^+^ matrix fibroblasts over total CD34^+^ are reduced in hyperoxia at PN4 and PN7 but are upregulated at PN10. (**E**) CD29^+^Ki-67^+^ myofibroblasts over total CD29^+^ are reduced in hyperoxia at PN4, unchanged at PN7, and upregulated at PN10. (**F**) CD29^+^CD34^+^Ki-67^+^ myo/matrix fibroblasts over total CD29^+^ CD34^+^ are reduced in hyperoxia at PN4, unchanged at PN7, and upregulated at PN10. In **D**–**F**, *n* = 4–6, control (RA) and experimental (O2) mice were used. A 2-tailed Student’s *t* test was used, *P < 0.05; **P < 0.01; ***P < 0.001. Error bars show mean ± SEM. (**G**) Schematic showing dynamic changes in the PDGFRA^+^ matrix fibroblast RNA signature gene expression and cell population in hyperoxia and RA recovery.

We have previously demonstrated that increased PDGFRA kinase activity promotes matrix fibroblast differentiation in PDGFRA^+^ fibroblasts (16). Based on scRNA-Seq data sets, the matrix fibroblast has the ECM as the dominant cellular component, with ECM organization and extracellular structure organization as hallmark GO signatures. Our gene expression analysis demonstrated a significant reduction of the matrix gene signature at all time points (Figure 1E). We generated a “developmental” matrix fibroblast signature gene list by extracting shared gene expression from the scRNA-Seq data set of matrix fibroblasts from PN7 and PN10 available on LGEA/LungMAP (51–53). The expression of matrix signature genes was compared between RA and hyperoxia fibroblasts at PN4, PN7, and PN10. RNA-Seq on sorted PDGFRA^+^ fibroblasts revealed select genes within the matrix fibroblast signature to be downregulated at PN4 (6 genes) and PN7 (8 genes) while others were unchanged (N/A) (Figure 3A). At PN10, 8 matrix fibroblast genes remained downregulated in hyperoxia, but 10 genes became upregulated in hyperoxia. Immunofluorescence for matrix fibroblast proteins fibronectin 1 (FN1) and cartilage associated protein (CRTAP) demonstrated parenchymal loss of both PDGFRA^+^FN1^+^ and PDGFRA^+^CRTAP^+^ matrix fibroblasts in the distal lung in hyperoxia at PN7 (Figure 3, B and C). To determine hyperoxia-mediated changes in matrix fibroblast function in PDGFRA fibroblasts, we performed flow cytometry on Lin^–^ fibroblasts and stained for PDGFRA^+^, CD29^+^, and CD34^+^ as previously described (16, 17) to discern between PDGFRA^+^ myofibroblasts (PDGFRA^+^CD29^+^) and PDGFRA^+^ matrix fibroblasts (PDGFRA^+^CD34^+^) (Supplemental Figure 5A). Proliferation was assessed by Ki-67^+^. Flow cytometry demonstrated a loss of proliferating CD34^+^Ki-67^+^ matrix fibroblasts among the PDGFRA^+^ population during PN4 and PN7 hyperoxia and significantly more proliferation during PN10 recovery (Figure 3D). There were significantly fewer Ki-67^+^ cells in the PDGFRA^+^CD29^+^ population at PN4, but more at PN10, indicating compensatory proliferation of myofibroblast population during hyperoxia recovery (Figure 3E). These data are comparable to the decrease in proliferation in PDGFRA^+^α-SMA^+^ cells (Figure 2B) and indicate that CD29 positivity precedes α-SMA expression. We previously identified the PDGFRA^+^CD29^+^CD34^+^ fibroblast as having characteristics of both myofibroblasts and matrix fibroblasts, which we denote here as myo/matrix fibroblasts (16, 17). During hyperoxia there were significantly fewer Ki-67^+^ myo/matrix fibroblasts among CD34^+^CD29^+^ cells at PN4 but more Ki-67^+^ myo/matrix fibroblasts at PN10 during recovery (Figure 3F). The RNA and population level matrix fibroblast changes are detailed in Figure 3G. Elastin gene expression is a signature of PN7 secondary crest fibroblasts (51). In hyperoxia lungs, elastin protein deposition in the tips of secondary crests was reduced, visualized by a Weigert’s Elastin stain (Supplemental Figure 5B).

### Hyperoxia-exposed PDGFRA^+^ fibroblasts fail to support alveolar organoid formation and AT1 cell differentiation in vitro.

**Figure 4:**
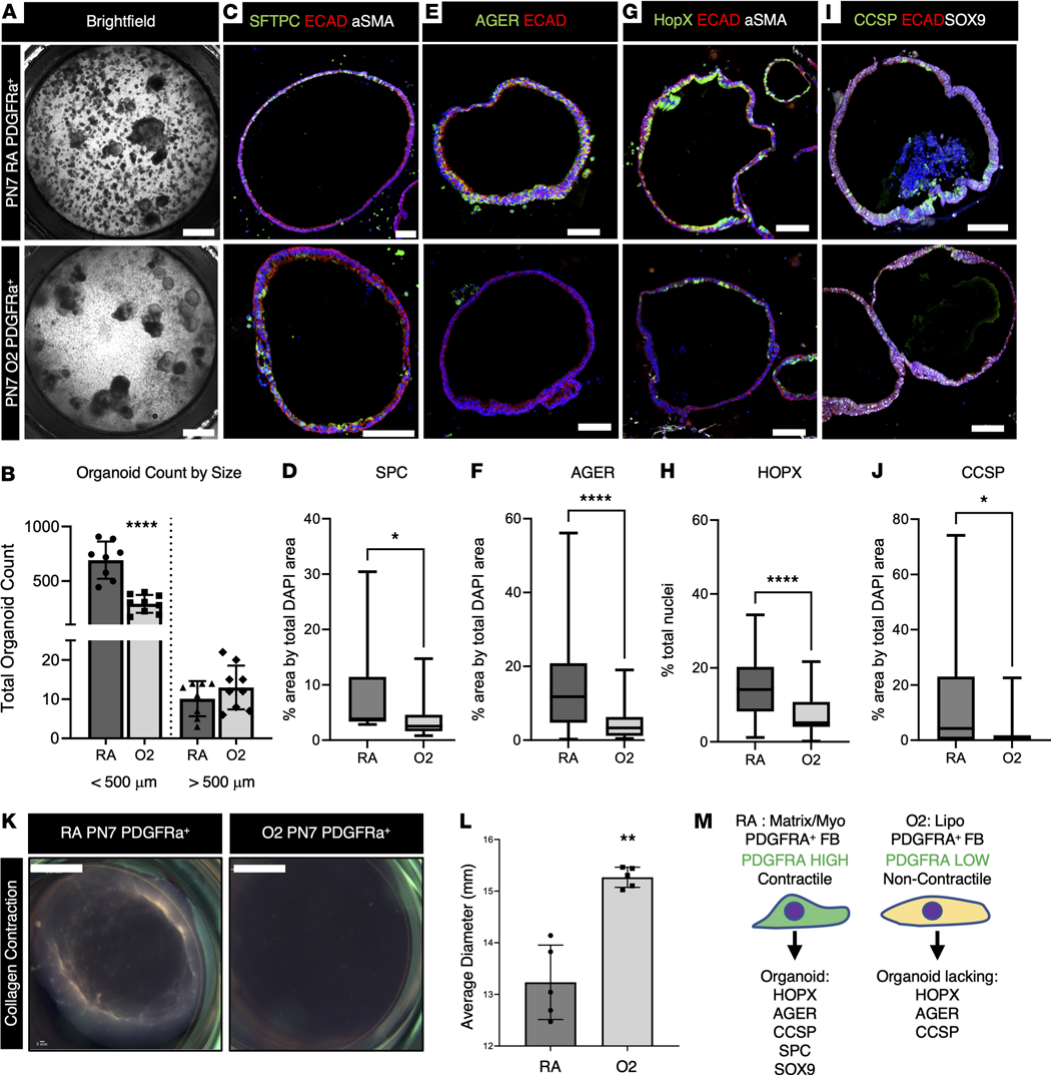
PDGFRA^+^ fibroblasts exposed to hyperoxia fail to support epithelial differentiation in organoid culture. (**A**) Organoids were made by coculturing fibroblasts from PN7 RA or O2-exposed (PN0–PN7, 90% O2) with adult RA epithelium, cultured for 3 weeks, and bright-field images were taken on fixed samples. Scale bar =1 mm. (**B**) Alveolar organoids were quantified on Nikon Elements by diameter, revealing reduced small colonies in hyperoxia. *n* = 8–10, control (RA) and experimental (O2) organoid transwells (replicates) were used. A 2-tailed Student’s *t* test was used, ****P < 0.0001. Error bars ± SD. (**C**,** E**, **G**, and **I**) Immunofluorescence on paraffin-embedded sections for markers of epithelial differentiation. Scale bars = 100 μm. (**D**, **F**, **H**, and **J**) Organoids were quantified using Nikon elements. Nuclear antibody stain HOPX was quantified by taking total antibody over DAPI, and cytoplasmic and membrane stains (SPC, AGER, CCSP) were quantified as % area over DAPI area. *n* = 8–10, control (RA) and experimental (O2) organoid transwells (replicates) were used. A 2-tailed Student’s *t* test was used, *P < 0.05; ****P < 0.0001. Data displayed as box-and-whisker plot. The box plots depict the minimum and maximum values (whiskers), the upper and lower quartiles, and the median. The length of the box represents the interquartile range. (**K**) Collagen contraction assay on PN7 PDGFRA^+^ RA and O2 fibroblasts was performed, then imaged after 3 days in culture. (**L**) Quantification of collagen assay average diameter in mm. Scale bar = 3.61 mm. Average diameter calculated, *n* = 5, RA and O2 collagen pellets were used. A 2-tailed Student’s *t* test was used, **P < 0.01. Error bars show mean ± SD. (**M**) Schematic showing how PN7 O2 PDGFRA^+^ fibroblasts fail to contract or support alveolar organoids in vitro.

We have shown that during hyperoxia injury and RA recovery, the PDGFRA^+^ fibroblast population was markedly reduced in overall fibroblast numbers and contained fewer myo/matrix fibroblasts (Figure 1G). How these functional changes in fibroblasts affect alveolar epithelial differentiation was assessed in organoid studies. MACS-isolated PDGFRA^+^ cells from PN7 hyperoxia or RA lungs were cultured with normal CD326-sorted epithelium from adult mouse lungs. After 3 weeks, organoid cultures were fixed, sectioned, and stained by IHC. Organoids were assessed for proximal and distal epithelial differentiation by expression for SFTPC, AGER, HOPX, CCSP, and SOX9. Hyperoxia-derived PDGFRA^+^ fibroblasts formed significantly fewer small organoids (Figure 4, A and B), suggesting a failure to support alveolar organoids in vitro. There was a small but significant decrease in SPC area over DAPI area, suggesting a reduction in the number of AT2 cells (Figure 4, C and D). Reduction in AGER area over DAPI area (Figure 4, E and F) and HOPX and DAPI dual-positive cells (Figure 4, G and H), indicated reduced AT1 differentiation. These findings are comparable to reduced AT2 and AT1 differentiation in vivo (Supplemental Figure 1F) (48). Unexpectedly, there was a significant reduction in club cell marker CCSP^+^DAPI^+^ cells, indicating that disrupted paracrine signals in the PDGFRA^+^ population may also affect proximal epithelial cell differentiation (Figure 4, I and J). There was no difference in P63^+^DAPI^+^ basal cells or SOX9^+^DAPI^+^ or SOX2^+^DAPI^+^ nuclei, which mark distal and proximal epithelial progenitors, respectively (Supplemental Figure 6, A–D). Taken together, these data demonstrate that hyperoxia-exposed PDGFRA^+^ fibroblasts fail to support epithelial differentiation in vivo and in vitro (Figure 4M).

### Loss of contraction in hyperoxia PDGFRA^+^ fibroblasts.

Contractile myofibroblasts are important for the elongation of the secondary crests (12, 16, 20, 54). MACS-isolated PDGFRA^+^ fibroblasts from PN7 RA and hyperoxia lungs were placed in a collagen pellet contraction assay (Figure 4, K and L). PDGFRA^+^ fibroblasts from hyperoxia mice contracted collagen pellets less efficiently than RA fibroblasts, indicating reduced commitment to the myofibroblast lineage (Figure 4, K and L). In summary, neonatal hyperoxia exposure increases the ratio of lipofibroblasts at the expense of myo/matrix fibroblasts within the PDGFRA^+^ fibroblast population. When isolated and placed in functional assays, these PDGFRA^+^ fibroblasts lose the ability to support alveolar organoid colony formation and AT1 cell differentiation, fail to support club cell differentiation, and are less contractile (Figure 4M).

### WNT signaling is selectively reduced in hyperoxia-exposed PDGFRA^+^ fibroblasts, and activation of WNT or PDGF-AA can restore matrix and myofibroblast function.

**Figure 5:**
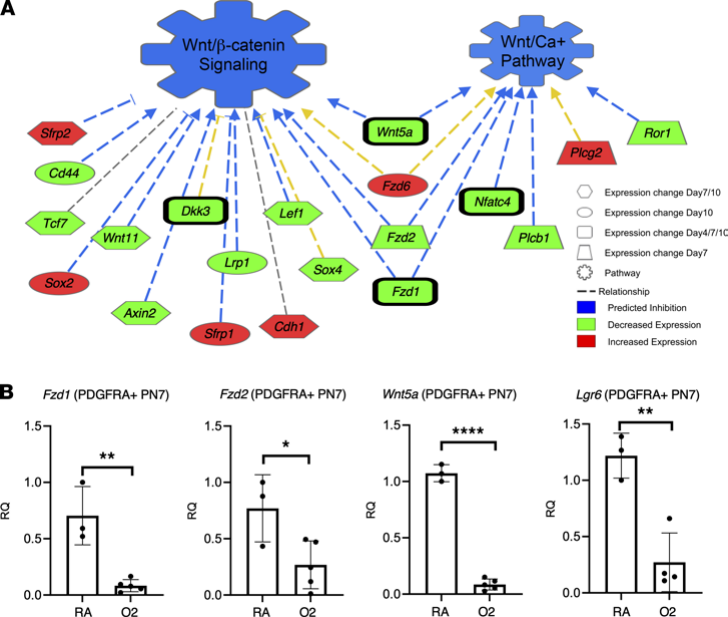
Gene expression changes in hyperoxia fibroblasts predict WNT signaling as an upstream regulator. (**A**) WNT-related gene changes and predictive network were generated by QIAGEN Ingenuity Pathway Analysis using genes significantly altered at PN4, PN7, and/or PN10 (87). (**B**) RT-qPCR on MACS-isolated PDGFRA^+^ fibroblasts validated expression of *Fzd1*, *Fzd2*, *WNT5a*, and *Lgr6*. *n* = 3–5, control (RA) and experimental (O2) mice were used. A 2-tailed Student’s *t* test was used, *P < 0.05; **P < 0.01; ****P < 0.0001. Error bars show mean ± SD.

**Figure 6:**
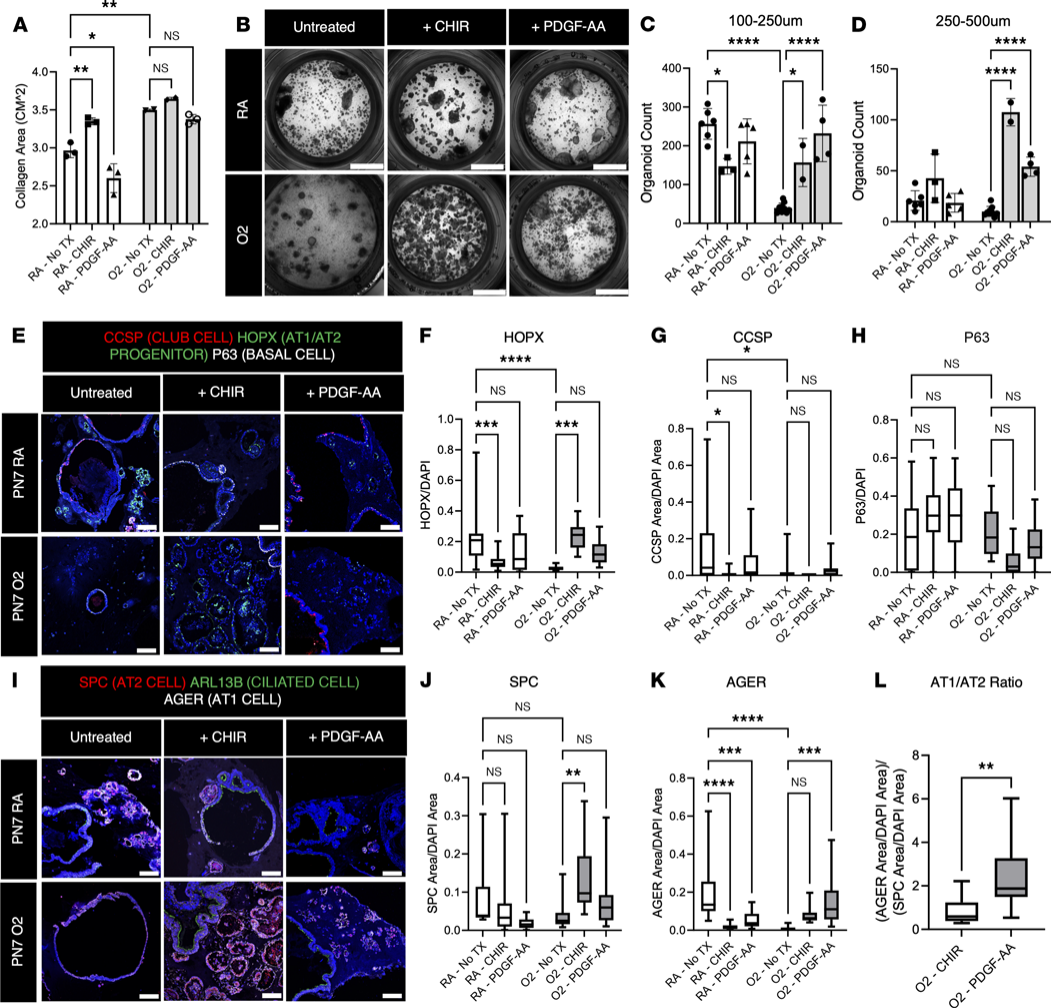
CHIR and PDGF-AA rescue support of the alveolar niche. (**A**) Collagen contraction assay on PN7 PDGFRA^+^ RA and O2 fibroblasts performed, treated with/without CHIR or recombinant hPDGF-AA (PDGF-AA), imaged after 2 days in culture, quantified by measuring collagen pellet area. *n* = 2–3 samples per group. (**B**) Organoids made by coculturing fibroblasts from PN7 RA or O2-exposed (PN0–PN7, 90% O_2_) with adult RA epithelium, treated with or without CHIR or PDGF-AA starting 7 days into culture. Bright-field images were taken on fixed samples after 3 weeks of growth. Scale bar = 2 mm. (**C** and** D**) Small alveolar and large alveolar organoids were quantified on Nikon Elements by restricting colony size to >100 μm and <250 μm in **C** and >250 μm and <500 μm in **D**. *n* = 2–10 Transwells (replicates). (**E** and **I**) Immunofluorescence on paraffin-embedded sections of organoids for markers of epithelial differentiation. Scale bar = 100 μm. (**F**–**H**,** J**, and** K**) Organoids quantified using Nikon Elements. Nuclear antibody stain (HOPX, P63) quantified by taking total antibody over DAPI, and cytoplasmic and membrane stains (SPC, AGER, CCSP) were quantified as antibody area over DAPI area. *n* = 2–10, organoid Transwells (replicates) used, 3 slides per Transwell. The box plots are as defined in Figure 4. In **A**,** C**,** D**,** F**–**H**,** J**, and** K**, 1-way ANOVA followed by Tukey’s multiple comparison was used to determine significance between 3 or more groups, *P < 0.05; **P < 0.01; ***P < 0.001; ****P < 0.0001. In **A**, **C**, and **D**, error bars show mean ± SEM. (**L**) Ratio of (AGER Area/DAPI Area)/(SPC Area/DAPI Area) within each image of O2 + CHIR versus O2 + PDGF-AA calculated to measure AT1/AT2 ratio. A 2-tailed Student’s *t* test was used, **P < 0.01. In **F**–**H** and **J**–**L**, data displayed as box-and-whisker plot.

Pathway analysis on the RNA-Seq data set predicted a substantial reduction of WNT signaling at all time points in hyperoxia-exposed PDGFRA^+^ fibroblasts (Figure 5A). We validated downregulation of the WNT pathway receptors *Lgr6*, *Fzd1*, and *Fzd2* and the WNT ligand *WNT5a* via RT-qPCR on MACS-isolated PN7 PDGFRA^+^ fibroblasts during hyperoxia (Figure 5B). These data suggest that hyperoxia PN7 PDGFRA^+^ fibroblasts are less receptive and responsive to incoming WNT signals. R-spondin signaling, which positively regulates WNT signaling, has recently emerged in BPD as a potential regulator of fibroblast differentiation (59). Human IMR90 lung fibroblasts are PDGFRA^+^ and derived from human fetal lung; thus, they are ideal to study molecular mechanisms that drive changes in fibroblast activation resulting in BPD (61). In vitro stimulation of human IMR90 PDGFRA^+^ neonatal lung fibroblasts with R-spondin–conditioned media increased expression of *TCF21* (Lipo), *FGF7* (mesenchymal alveolar niche cell [MANC]), and *CD248* (Myo) signature genes (Supplemental Figure 4A). However, R-spondin treatment of hyperoxia fibroblast organoids did not significantly rescue organoid formation or epithelial differentiation (data not shown). In contrast, the canonical WNT activator CHIR (62) reduced expression of *CD248*, *FGF7*, and *WNT2* and induced *AXIN2* and *WNT5A* expression in IMR90 human fibroblasts and AXIN2 in both RA and hyperoxia fibroblasts (Supplemental Figure 4, B–D). We assessed myofibroblast differentiation by CHIR and PDGF-AA treatment in PN7 RA and hyperoxia PDGFRA^+^ fibroblasts in a collagen contraction assay. Interestingly, CHIR treatment of RA fibroblasts reduced collagen contraction while PDGF-AA increased contraction. In hyperoxia PDGFRA^+^ fibroblasts, neither CHIR nor PDGF-AA could restore myofibroblast contractions (Figure 6A). These data suggest that WNT and PDGFA activation do not change myofibroblast differentiation.

### Activation of WNT or PDGFRA signaling restores lung organoid formation and AT2/AT1 differentiation.

We have previously published that activation of matrix fibroblast function by treatment with PDGF-AA ligand restoreS AT1 and AT2 differentiation in human idiopathic pulmonary fibrosis (IPF) lung organoids (37). To assess whether activation of canonical WNT signaling with CHIR or matrix fibroblast function with PDGF-AA restores alveolar organoid formation in hyperoxia fibroblasts and their ability to support AT2/AT1 differentiation, we cultured organoids made with PN7 RA or hyperoxia fibroblasts and adult alveolar epithelial cells in the presence of CHIR or PDGF-AA (Figure 6, B–D, and Supplemental Figure 6F). CHIR treatment of RA fibroblast organoids decreased the number of small alveolar organoids (<250 μm) and increased the number of larger organoids, while PDGF-AA treatment did not change the number or size of RA organoids. Hyperoxia fibroblasts had a significant reduction (*P* < 0.0001) in small organoid formation, which was restored by CHIR and PDGF-AA treatment. Large organoids made with hyperoxia fibroblasts were also markedly increased by both CHIR and PDGF-AA treatment. In addition, CHIR-treated organoids formed tubular structures that were not observed before. These data demonstrate that CHIR and PDGF-AA treatment restores lung organoid formation and growth in hyperoxia PDGFRA^+^ fibroblasts and that hyperoxia fibroblasts are more susceptible to stimulation by canonical WNT signaling.

**Figure 7:**
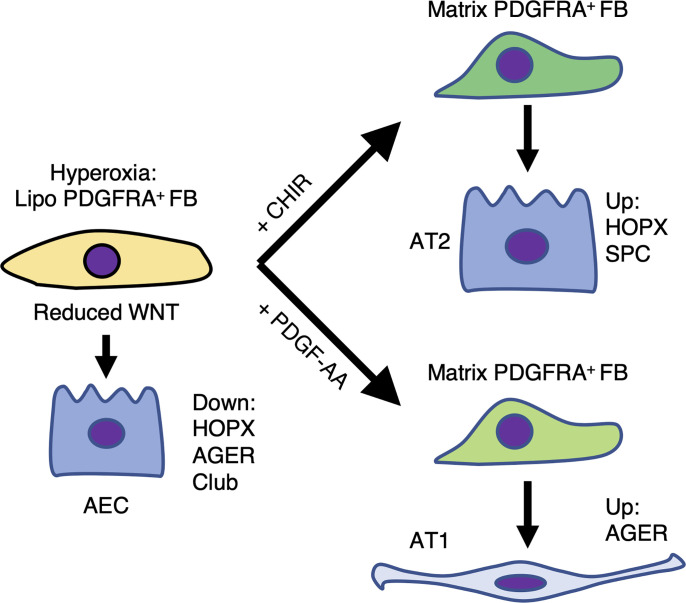
Model. In vivo hyperoxia exposure results in increased PDGFRA^+^ lipofibroblast differentiation. Reduced WNT signaling emanating from lipofibroblasts results in downregulation of AT1 and club cell differentiation. CHIR treatment of lung organoids made with hyperoxia fibroblasts restores AT2 cell (SPC) differentiation and AT1 progenitors (HOPX). PDGF-AA treatment of lung organoids made with hyperoxia fibroblasts restores AT1 differentiation (AGER).

We assessed epithelial differentiation by immunofluorescence for HOPX, AGER, SPC, CCSP, ARL13B, and P63 (Figure 6, E–K, and Supplemental Figure 6E). In RA organoids, CHIR reduced HOPX, AGER, and CCSP expression. In hyperoxia organoids, CHIR increased the total number of SPC^+^ cells and rescued the number of HOPX^+^ AT1 progenitors. These data suggest that CHIR reduces the ability of RA fibroblasts to drive epithelial differentiation but activates both expansion of AT2 cells and AT1 progenitor cells in hyperoxia fibroblasts. In contrast, PDGF-AA treatment of RA organoids decreased AGER expression, but in hyperoxia organoids, increased AGER. The ratio of AGER to SPC protein expression was significantly higher in PDGF-AA–treated hyperoxia organoids compared with CHIR-treated organoids, indicating that PDGF-AA directs the mesenchyme to support AT2-to-AT1 conversion, while CHIR directs the mesenchyme to support AT2 proliferation (Figure 6L). These results are summarized in Figure 7, where PN7 fibroblasts from hyperoxia-exposed lungs retain their plasticity and can be pushed to support distal epithelial differentiation through treatment with either PDGF-AA or CHIR.

## Discussion

Neonatal care has progressed to a point where lifesaving interventions such as antenatal corticosteroids, surfactant replacement, and mechanical ventilation can stabilize a premature infant’s lungs. Due to a poor understanding of maladaptive alveolarization in BPD pathogenesis, it remains unclear how to harness and augment normal mechanisms of alveolarization following an injury to fully restore lung function (29, 63–65). While other research groups have focused on lineage relationships to identify the progenitor cell of the fibrotic fibroblasts in hyperoxia, we try to dissect phenotypical changes in a population that is defined by expression of PDGFRA. PDGFRA-expressing fibroblasts are very likely derived from different lineages, as PDGFRA expression is activated in a dynamic and spatial/temporal manner (12, 15–17, 20, 54, 66). Fibroblasts support epithelial and endothelial cells and very likely react to injury in a context-dependent manner. In this study, we focused on functional changes of PDGFRA^+^ fibroblasts and proportional changes of myo-, lipo-, and matrix fibroblasts within the general PDGFRA^+^ population. Within the PDGFRA^+^ fibroblasts, we identified loss of contractility in myofibroblasts, reduced ECM remodeling in matrix fibroblasts, and an increase in lipofibroblast differentiation during hyperoxia injury that persisted during the RA recovery phase. We linked matrix fibroblast function with WNT signaling and provide evidence that matrix fibroblasts are essential for proper AT1 differentiation.

Excessive myofibroblast differentiation has been documented in lungs after hyperoxia injury (59). Here we demonstrate that PDGFRA^–^ myofibroblasts increase during hyperoxia injury and very likely represent the myofibroblast population previously documented to expand (30, 67). These data are in agreement with a murine hyperoxia study, where the PDGFRA^+^ population was lineage-traced and shown not to contribute to hyperoxia-associated myofibroblasts (12). It has been well documented that the secondary crest fibroblasts are important for septal elongation and loss of transient myofibroblast differentiation during secondary septation results in alveolar simplification (9, 13, 15, 20, 23, 68, 69). In this study, we identified a loss of PDGFRA^+^ myofibroblasts during hyperoxia injury and a moderate compensatory increase during recovery. Moreover, we showed that PN7 hyperoxia-exposed PDGFRA^+^ fibroblasts demonstrated reduced collagen contractility, supporting previous findings that contractile myofibroblasts are important for septation (12). These data suggest that hyperoxia impedes PDGFRA^+^ myofibroblast differentiation during a critical developmental window which results in suppressed septation and alveolar simplification in BPD.

We saw a consistent expansion of PDGFRA^+^ lipofibroblasts during hyperoxia injury, which diverges from classical reports of the lipofibroblast in hyperoxia, which did not distinguish between PDGFRA-positive and -negative fibroblasts (30, 36). Previous in vivo and in vitro studies describe an overall loss of lung lipofibroblasts after hyperoxia (30, 36). In contrast, here we report that hyperoxia increased the PDGFRA^+^ lipofibroblast population. Lipofibroblasts are known to support AT2 cells; this supportive role was maintained in hyperoxia lungs as we did not find any changes in AT2 cell differentiation in vivo (34, 40, 68, 70, 71).

We recently showed that the PDGFRA^+^ matrix fibroblast is lost in nonfibrotic areas of IPF lungs and that PDGFRA fibroblasts in aged lungs are committed to a myofibroblast phenotype (37). In vitro organoid cocultures with aged PDGFRA fibroblasts resulted in a decreased number of organoids and failed AT1 differentiation (8, 72). In this hyperoxia study, we find that the loss of the PDGFRA^+^ matrix fibroblast signature is correlated with a similar decrease in the number of organoids and failed AT1 differentiation. Many of the developmental matrix fibroblast signature genes (51) overlapped with signature genes of the adult MANC, including *Fgf7*, *Pdgfra*, and *WNT2* (19, 51, 73). MANCs and PDGFRA^+^ fibroblasts are known to support the alveolar niche in adult lungs and have been shown to support AT2-to-AT1 transdifferentiation in lung organoid cultures (10, 73, 74). These data suggest that the developmental matrix fibroblast is very similar to the regenerative adult MANC. Future studies dissecting adult and developmental PDGFRA^+^ fibroblasts combined with lineage tracing experiments might reveal a unique fibroblast progenitor population that gives rise to both developmental secondary crest myofibroblasts and regenerative adult MANCs.

WNT signaling has been implicated in epithelial self-renewal, epithelial differentiation in adult injury repair, and 3D cell culture models (73, 75, 76). However, sustained WNT signaling has been associated with fibrotic injury response and IPF (77–81). Several studies looked at the induction of WNT signaling in the lung mesenchyme and epithelium after hyperoxia injury and found that inhibiting WNT can rescue hyperoxia-induced injury (59, 82, 83). In this study, we report that hyperoxia exposure in vivo results in gene expression and functional changes in PDGFRA^+^ fibroblasts that are predicted to be downstream of WNT signaling. The functional changes in PDGFRA^+^ fibroblasts resulted in impaired support of AT2 and AT1 differentiation in vivo and in organoid studies. Activation of canonical WNT signaling by CHIR treatment of human fetal PDGFRA^+^ fibroblasts (IMR90) resulted in downregulation of *FGF7* and upregulation of *AXIN2*, which would result in a profibrotic fibroblast activation (73). CHIR treatment of RA versus hyperoxia organoids had the opposite effect. Our data suggest that RA fibroblasts react to canonical WNT signaling by becoming less supportive of epithelial differentiation and more profibrotic, whereas hyperoxia fibroblasts react to canonical WNT activation with increased regenerative and niche-supporting functions. All these studies demonstrate context-dependent, cell-specific beneficial and detrimental WNT function during normal development and disease pathogenesis. Recent studies highlight the matrix fibroblast as an important cell in IPF (37), supporting a similar important role in BPD pathobiology. Future studies on the specific role of matrix fibroblasts during early lung development will reveal whether pharmacological induction of matrix fibroblast differentiation by WNT signaling modulators will have beneficial effects on alveolar septation and the epithelial differentiation in BPD and IPF.

## Methods

Additional materials and methods are provided in Supplemental Methods.

### Murine neonatal hyperoxia exposure.

Newborn WT C57BL/6J mice (from mice from The Jackson Laboratory) were placed in either RA or into a hyperoxia O_2_ (O2) 30″ × 20″ × 20″ propylene chamber in which the oxygen concentration was maintained at 90% O_2_. For injury time points, pups were continuously exposed to hyperoxia from PN0 until being harvested at PN4 or PN7. For recovery time point, neonatal mice were exposed to 90% O_2_ from PN0–PN7 then returned to RA until being harvested at PN10. Nursing dams were rotated between groups every 24 hours to prevent oxygen toxicity damage to the dams.

### Cell isolation, RNA purification, and quantification.

PDGFRA^+^ cells were isolated from homogenized lungs by positive selection using MACS (Miltenyi Biotec) following manufacturer’s instructions. Epithelial cells were isolated with Anti-Mouse CD326 (EpCAM) MicroBeads, and fibroblasts were isolated with Anti-Mouse CD140A (PDGFRA) MicroBeads (Miltenyi Biotec). RNA was extracted from MACS-sorted cells or cells harvested from IMR90 cell culture using the RNeasy Mini Kit (QIAGEN, 74104). RNA quality was verified using a spectrophotometer (NanoDrop, Thermo Fisher Scientific). RNA was reverse-transcribed into single-stranded cDNA using the Verso complementary DNA synthesis kit (Life Technologies, AB-1453). Quantitative PCR was conducted using TaqMan primers and TaqMan Master Mix (Life Technologies) and measured using StepOnePlus Real Time PCR system (Thermo Fisher Scientific) (see Supplemental Table 2 for primer information). Bulk RNA-Seq was performed on MACS-isolated PDGFRA^+^ cells from PN4, PN7, and PN10 neonatal mice by Cincinnati Children’s Hospital Medical Center’s Gene Expression Core (GEO accession GSE171812; see Supplemental Table 5).

### Flow cytometry staining.

Freshly isolated mouse lung cells (*n* = 4–6) from PN4, PN7, and PN10 were washed and resuspended in staining buffer (Hanks 1× PBS, 1 mM EDTA, 25 mM HEPES, 2% FBS) and then blocked for 30 minutes with anti-CD16/32 (eBioscience). Cells were washed with staining buffer and then incubated with fluorescently conjugated surface antibodies on ice for 30 minutes; they were then subsequently fixed, permeabilized, and stained with intracellular antibodies (Supplemental Table 3). To set positive gates and compensate for the overlap of fluorescence emission spectra, fluorescence minus one and fluorescent antibodies were used (Supplemental Table 4). Side scatter pulse-width with forward scatter pulse-height and forward scatter pulse-width with side scatter pulse-height excluded doublets; dead cells were excluded using the viability dye. Cells were sorted with a BD LSR II analyzer with 5 lasers, and the results were analyzed using FlowJo software.

### Organoids.

Pups from 2 or more litters of WT C57BL/6J were exposed to RA and hyperoxia as described above for 7 days and then harvested on PN7 for organoid culture. An adult mouse from the same line kept in RA was harvested for epithelial cells. After MACS for PDGFRA^+^ fibroblasts (RA and hyperoxia pups) and CD326^+^ EPI (RA adult), cells were combined in a 10:1 ratio (FB/EPI) and mixed with Cultrex basement membrane extract (Trevigen BME; laminin, coll-iv, entactin, heparan sulfate proteoglycan) at a 1:1 ratio. The mixture was loaded in air-liquid-interface Transwells on a 24-well plate (both from Falcon), cultured in DMEM-Ham’s F12 media (Gibco) with added nutrients (5% FBS, 15 mM HEPES, penicillin and streptomycin, fungizone, 10 μg/mL insulin, 5 μg/mL transferrin, 0.1 μg/mL cholera toxin, 25 ng/mL EGF, and 30 μg/mL bovine pituitary extract) for 21 days, and fixed for immunofluorescence (see Supplemental Table 1 for antibodies used in the study).

### Statistics.

An unpaired Student’s 2-tailed *t* test was used to determine the significance between 2 groups. A 1-way ANOVA followed by Tukey’s multiple-comparison test was used to determine significance between 3 or more groups. GraphPad Prism 9.0.0 was used to calculate statistical differences and for creation of the associated graphs. Graphs are presented as dot plots with the mean as the top of the underlying bar graph, and error bars are ± SEM or ± SD, specified in the figure legends. *P* < 0.05 was considered statistically significant, and levels of significance were denoted as *P < 0.05, **P < 0.01, ***P < 0.001, ****P < 0.0001.

### Study approval.

Mice are housed in a pathogen-free facility accredited by the Association for Assessment and Accreditation of Laboratory Animal Care at Cincinnati Children’s Hospital Medical Center (CCHMC). All animal protocols used in this study were approved by the Institutional Animal Care and Use Committee at CCHMC. No human studies were performed, and no human samples were used.

## Author contributions

MRR, MGU, and MW are co–first authors; they each had lead roles in designing and performing experiments and performing data analysis. MRR is listed first because of his lead role in writing the manuscript and designing and performing experiments critical to the organoid cultures; MGU is listed second because of her contributions to experiments and the manuscript; and MW is listed third because of her lead role in the early project design and experimentation. JG performed and analyzed the organoid experiment. SED performed functional enrichment analysis on the RNA-Seq data and generated the associated heatmap. JS generated the signature gene heatmaps (51, 84), created the WNT predictive network, and edited the manuscript. BL performed the R-spondin treatment on IMR90 cells. ME performed the flow cytometry experiments. SKA, JW, and AKTP designed experiments, interpreted data, and edited the manuscript. AKTP cowrote the manuscript.

## Supplementary Material

Supplemental data

## Version 1 02/03/2022

In-Press Preview

## Version 2 03/08/2022

Electronic publication
