# Supplementary material for: Maladaptive functional changes in alveolar fibroblasts due to perinatal hyperoxia impair epithelial differentiation
Source: JCI Insight. 2022 Mar 8;7(5):e152404. doi: 10.1172/jci.insight.152404 (PMC8983125; doi:10.1172/jci.insight.152404)
Supplement: Supplemental data [file jciinsight-7-152404-s240.pdf]

Supplemental Materials and Methods

Mouse housing and euthanasia information

Mice were maintained on campus at the CCHMC animal facility. Mice were on a 12hrs:12hrs dark/light cycle and received food and water ad libitum. Pups were euthanized with 50 ul of triple sedative solution (67mg/ml ketamine + 3.3 mg/ml xylazine + 1.7 mg/ml acepromazine) by intraperitoneal injection and a method of secondary euthanasia was employed (exsanguination by severing the carotid artery). Adult mice used in the organoid study were euthanized in the same manner but received 100 ul of triple sedative solution.

Collagen Contraction Assay

Collagen pellets were generated following previously established protocols (1-3). Collagen pellets were prepared in a 12 well tissue culture plate that was coated with 1% BSA in media and incubated for 2 hrs at 37°C. After removal of media, 1ml of 1 mg/ml type I collagen (BD Biosciences) (Figure 4 K) or 0.4mg/ml (Figure 6 A) was added and allowed to polymerize for 16 hrs at 37°. The resulting collagen matrix was detached from the sides of the well using a sterile 10ul pipette tip. PDGFRA^{+} cells from PN7 room air and hyperoxia neonatal mice were seeded on the free-floating collagen matrix at 100,000 cells in 10% FBS/DMEM Hams F12; 0.1% penicillin and streptavidin; 0.1% gentamycin and amphotericin in triplicate. Pellets were cultured for 3 days (Figure 4 K) or 24hrs with or without 5 µM CHIR or 10 ng/ml PDGF-AA (Figure 6 A) and then imaged using a Leica DFC7000 T camera attached to a Leica MZ16 FA fluorescent stereomicroscope.

Contraction was calculated with Image J software by measuring the diameter of the pellet, followed by a 2-tailed Student's t-test (Figure 4 L) or ANOVA followed by Tukey's multiple comparison (Figure 6 A).

RNA-seq and bioinformatic analysis

MACS-sorted PDGFRA$^+$ (CD140$^+$) fibroblasts from either RA or O_2 PN4, PN7, and PN10 mouse lungs were prepared for bulk RNA-seq. RNA sequencing was performed by Cincinnati Children's Hospital Medical Center's Gene Expression Core utilizing the Illumina HiSeq2500. RNA-seq FASTQ files were aligned using Bowtie to mouse genome version mm10 (4). Raw gene counts were obtained using Bioconductor's Genomic Alignment, which were subsequently made into normalized FPKM values using Cufflinks (5, 6). DeSeq (Bioconductor) was used to calculate differential gene expression from raw expression values. Genes were deemed differentially expressed if they satisfied the following requirements: gene has a fold change >2, binomTest pvalue <.01, and RPKMs >1 in 2 of the 3 replicates in at least one condition being compared. Gene patterns were determined by comparing differential expressed genes from all three time points. Genes that were significantly changed or unchanged at the same time points were grouped together. Heatmaps of genes in particular patterns were z-score normalized and generated using Partek Genomics Suite (http://www.partek.com/pgs). Gene enrichment analysis was carried out using ToppGene's ToppFun, and functional enrichments within each profile were identified and all profiles were compared to each other using Toppcluster (7). Pvalues of functional enrichment hits where $-\log_{10}$ transformed for graphical visualization. Signature genes for Matrix and Myo fibroblast were determined

by downloading and comparing PN7 and PN10 markers genes for respective cell types from LGEA https://research.cchmc.org/pbge/lunggens/mainportal.html (8). Only signatures genes identified at both time points for a particular cell type were assessed. Top 50 Lipofibroblast signature genes were obtained from a recently published mouse lung scRNA-seq study (9). Fold changes of any Matix, Myo or Lipo fibroblast marker genes significantly altered for a particular cell type, at any time point, were visualized in a heatmap generated by Pheatmap (https://cran.r-project.org/web/packages/pheatmap/index.html). Wnt related genes changes and predictive network was generated by Qiagen Ingenuity Pathway Analysis (IPF) using genes significantly altered at D4, D7 and/or Day10 (10).

Immunofluorescence

Immunofluorescent staining was performed on 5-μm slides sectioned from paraffin-embedded lung tissue blocks. Slides were deparaffinized in xylene, rehydrated in a series of graded ethanol and washed in 1X PBS. When required, antigen retrieval in 10 mM citrate buffer (pH 6.0) was performed. Non-specific antigens were blocked in 4% normal donkey serum in PBS with 0.1% Triton X-100 (PBST) for 2 hours. Slides were incubated in primary antibodies (Supplemental Table 1) diluted in blocking buffer overnight at 4°C. After washing in PBST, slides were incubated in fluorescent secondary antibodies (1:200) and DAPI (1μg/ml) diluted in blocking buffer for 1 hour at room temperature. Slides were subsequently washed in PBST and mounted in Prolong Gold (Thermo Fisher Scientific). Z-stack images were captured on a Nikon AR1 inverted confocal microscope and further

analyzed using Nikon Elements software. Antibody staining was quantified on Nikon Elements using a previously established protocol (11).

In vitro r-spondin treatment of IMR90 cells

Human IMR90 fibroblasts (ATCC® CCL-186™) were cultured in growth medium (DMEM Hams F12; 10% FBS; 0.1% penicillin and streptavidin; 0.1% gentamycin and amphotericin) until passage 3 in a 100mm tissue culture plate. Cells were seeded on a 24-well plate, and upon reaching 70% confluency, treated with r-spondin conditioned media at a dilution of 1:500. Cells were harvested at 12, 24, and 36 hrs after r-spondin treatment and processed for RNA isolation and RT-qPCR gene expression analysis using the same methods as previously described.

Morphometrics

Morphometric analysis with a FIJI-macro was used to quantify alveolar structure including volume density of alveolar septa (Vv_{sep}), mean linear intercept of airspaces (L*m*), mean transsectional wall length (L*mw*), and surface area density of airspaces (Sv_{air}) on PN4, PN7 and PN10 room air and hyperoxia exposed mouse lungs. Lungs were inflation-fixed and processed as previously described and 7-µm thick sections were stained with hematoxylin and eosin for analysis. From each time point and treatment group (N = 3), five sections with the whole lung were imaged at 10X collecting five to seven random pictures of each section. The FIJI macro uses test lines to automatically count the number of points landing on tissue and the number of intersections of air-tissue interface (). Any

non-parenchymal tissue or large vessels and conducting airways were manually selected to be excluded from the analysis. The values collected for each section were averaged and used as a data point in the final morphometric representation.

CHIR99021 and PDGF-AA treatment of IMR90 cells and primary mouse cultures

Human IMR90 fibroblasts and primary mouse MACS-isolated PN7 PDGFRA$^+$ fibroblasts in monolayer were treated in monolayer with 5 µM WNT activator CHIR99021 (Tocris, cat # 4423, CHIR). Human IMR90 fibroblasts and primary mouse MACS-isolated PN7 PDGFRA$^+$ fibroblasts in collagen contraction and organoid assays were treated in media with either 10 ng/ml recombinant human hPDGF-AA (R + D Systems, cat # 221-AA-010, PDGF-AA) or 5 µM CHIR. CHIR was diluted in DMSO to a 2mM stock concentration and then serially diluted in media to a working concentration of 5 µM. PDGF-AA was reconstituted at 100µg/mL in sterile 4mM HCL, and then serially diluted to 10 ng/ml in media as a working concentration. IMR90 fibroblasts and primary mouse PN7 PDGFRA$^+$ FBs were treated for 24hrs continuously with CHIR after plating. Primary fibroblasts in collagen contraction assays were treated for 24hrs continuously with CHIR or PDGF-AA after seeding on top of collagen. Organoids were allowed to grow for 7 days before treatment with either CHIR or PDGF-AA added to the organoid media, continuously for 14 days (with media changes every two days).

Supplemental Table 1
List of primary antibodies used in this study

**Immunofluorescent Antibody**	**Source**	**Catalog #**	**Dilution**	**Antigen Retrieval**
aSMA	Abcam	A5228	1:10,000	Citrate
ADRP	Abcam	Ab52356	1:200	Citrate
PDGFRA	R+D Systems	AF1062	1:50	Citrate
FN1	Abcam	Ab23750	1:100	Citrate
CRTAP	Gift from Dr. Roy Morello	N/A	1:200	Citrate
SOX9	Millipore	AB5535	**1:200**	Citrate
SOX2	Seven Hills Bioreagents	WRAB-1236	**1:200**	Citrate
CCSP	Seven Hills	G210	1:3000	Citrate
ECAD	R+D Systems	MAB7481	1:2000	None
AGER	R+D Systems	AF1145	1:200	Citrate
HOPX	Santa Cruz	Sc-30216	1:100	Citrate
SPC	Seven Hills	R458	1:100	Citrate
ARL13B	Proteintech	17711-1-AP	1:100	Citrate

Supplemental Table 2
List of Taqman primers used in this study

**Gene**	**TaqMan probe catalog #**
Pdgfra	Mm00440701
Fzd1	Mm00445405
Fzd2	Mm02524776
Wnt5a	Mm00437347
Lgr6	Mm05916284
Axin2	Mm00443610
Pdgfa	Mm01205760
Hopx	Mm00558639
AXIN2	Hs00610344
TCF21	Hs00162646
CD248	Hs00535586
FGF7	Hs00940253
WNT2	Hs00608224
WNT5A	Hs00998537

Supplemental Table 3
Flow cytometry primary antibodies used in this study

Flow Cytometry Antibody	Source	Catalog #	dilution
aSMA	Sigma	A5228	1/10,000
CD31 (PECAM-1)	E-Biosciences	17-0311	1/100
	E-Biosciences	48-0311	1/100
CD34	E-Biosciences	50-0341	1/100
	E-Biosciences	48-0341	1/100
CD45	E-Biosciences	48-0451	1/100
	E-Biosciences	17-0451	1/100
CD140a (PDGF Receptor a)	E-Biosciences	17-1401	1/100
CD326 (EpCAM)	E-Biosciences	48-5791	1/100
Ki-67	E-Biosciences	51-5698	1/100
CD29	E-Biosciences	47-0291	1/100
ADRP	abcam	ab108323	1/250
Live/Dead	E-Biosciences	65-0865-14	1/1000

Supplemental Table 4
Flow Cytometry Panel Design

**Laser**	PE	PE-Cy7	AF594	AF488	PerCP cy5.5	Efluor450	APC	AF700	APC Cy7	UV
**filter**	**565-605**	**760-780**	**600-620**	**425-475**	**655-730**	**425-475**	**655-730**	**710-750**	**760-780**	**425-475**
FMO-1	-	CD326	αSMA	ADRP	CD45	Ki-67	CD31	CD34	CD29	Live/Dead
FMO-2	CD140a	_	αSMA	ADRP	CD45	Ki-67	CD31	CD34	CD29	Live/Dead
FMO-3	CD140a	CD326	_	ADRP	CD45	Ki-67	CD31	CD34	CD29	Live/Dead
FMO-4	CD140a	CD326	aSMA	_	CD45	Ki-67	CD31	CD34	CD29	Live/Dead
FMO-5	CD140a	CD326	αSMA	ADRP	-	Ki-67	CD31	CD34	CD29	Live/Dead
FMO-6	CD140a	CD326	αSMA	ADRP	CD45	_	CD31	CD34	CD29	Live/Dead
FMO-7	CD140a	CD326	αSMA	ADRP	CD45	Ki-67	_	CD34	CD29	Live/Dead
FMO-8	CD140a	CD326	αSMA	ADRP	CD45	Ki-67	CD31	_	CD29	Live/Dead
FMO-9	CD140a	CD326	αSMA	ADRP	CD45	Ki-67	CD31	CD34	_	Live/Dead
FMO10	CD140a	CD326	αSMA	ADRP	CD45	Ki-67	CD31	CD34	CD29	_

Supplemental Table 5
Geo accession information for RNA-seq files

GEO Submission #	Project			
GSE171812	Maladaptive functional changes in alveolar fibroblasts due to perinatal hyperoxia impair epithelial differentiation			
**Accession**	**Title**	**Releasee Date**	**Status**	**Supplementary Files**
GSM5234590	SA2017_109	3-Nov-21	Approved	None
GSM5234591	SA2017_110	3-Nov-21	Approved	None
GSM5234592	SA2017_111	3-Nov-21	Approved	None
GSM5234593	SA2017_112	3-Nov-21	Approved	None
GSM5234594	SA2017_132	3-Nov-21	Approved	None
GSM5234595	SA2017_133	3-Nov-21	Approved	None
GSM5234596	SA2017_134	3-Nov-21	Approved	None
GSM5234597	SA2017_137	3-Nov-21	Approved	None
GSM5234598	SA2017_138	3-Nov-21	Approved	None
GSM5234599	SA2017_139	3-Nov-21	Approved	None
GSM5234600	SA2017_17	3-Nov-21	Approved	None
GSM5234601	SA2017_19	3-Nov-21	Approved	None
GSM5234602	SA2017_39	3-Nov-21	Approved	None
GSM5234603	SA2017_40	3-Nov-21	Approved	None
GSM5234604	SA2017_41	3-Nov-21	Approved	None
GSM5234605	SA2017_42	3-Nov-21	Approved	None
GSM5234606	SA2017_57	3-Nov-21	Approved	None
GSM5234607	SA2017_58	3-Nov-21	Approved	None

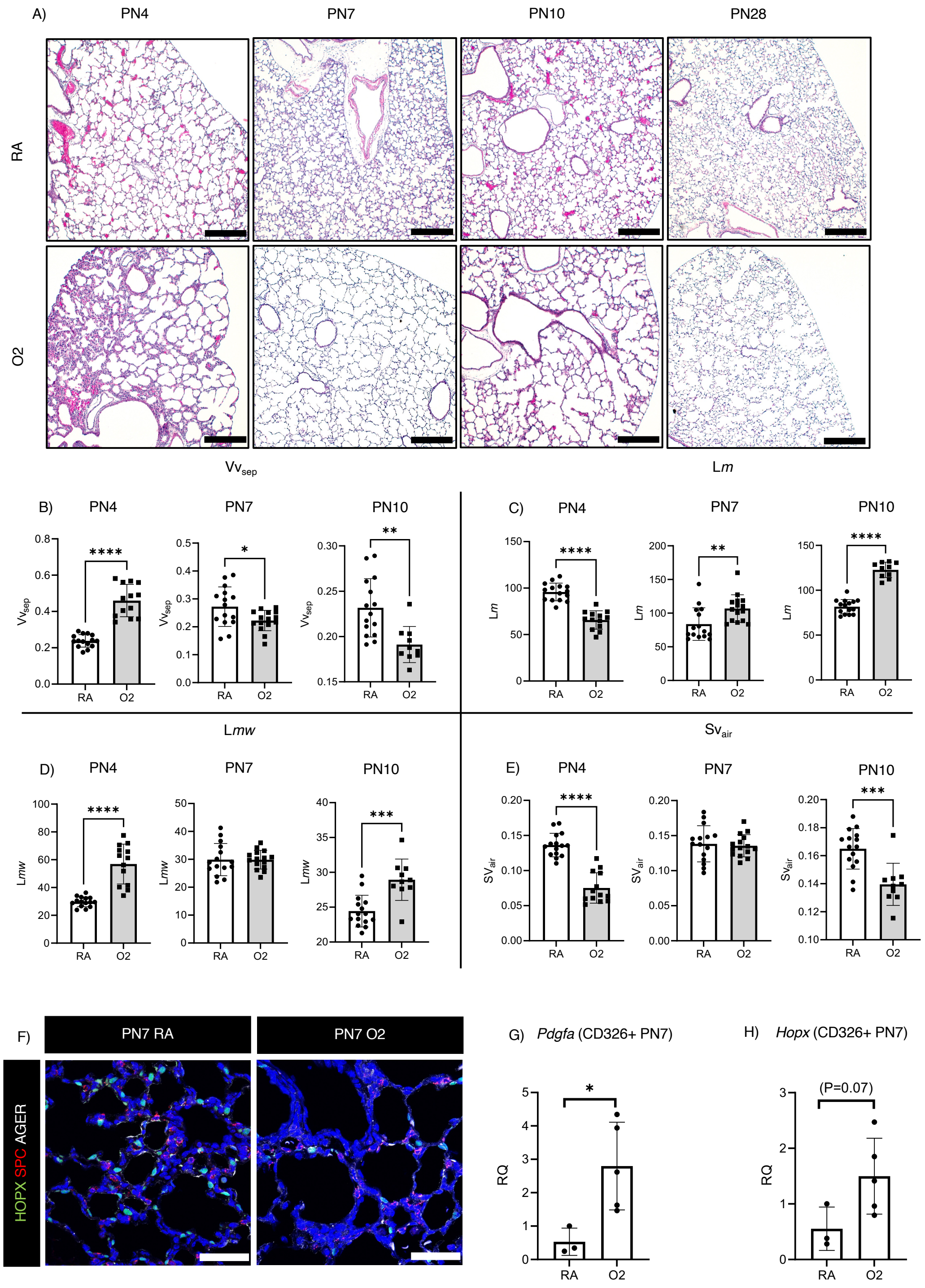

Supplemental Figure 1. Changes to murine lung morphology due to hyperoxia during injury and early repair
A. H + E staining of PN4, PN7, PN10, and PN28 RA and O2 lungs. Scale bar = 250 µm. **B-E.** Vv_{sep} (Volume density of alveolar septa), L*m* (Mean linear intercept), L*mw* (mean transactional wall length), Sv_{air} (Surface area density of airspaces) of PN4, PN7, and PN10 RA and O2 H + E images, N=3 mice per group, 5 slides per mouse. A 2-tailed Student's t test was used, *P<0.05, **P<0.01, ***P<0.001. ****P<0.0001, error bars ± SD **F**. Immunostaining of PN7 RA and O2 fixed lungs reveals reduced HOPX staining but no noticeable difference in SPC or AGER. Scale bar=50 µm. **G, H**. *PDGFA* and *HOPX* RT-qPCR on CD326+ epithelial cells, isolated by MACS, from room air- and hyperoxia-exposed PN7 mouse lungs. In panels (C, D), a 2-tailed Student's t test used, *P<0.05, error bars ± SD.

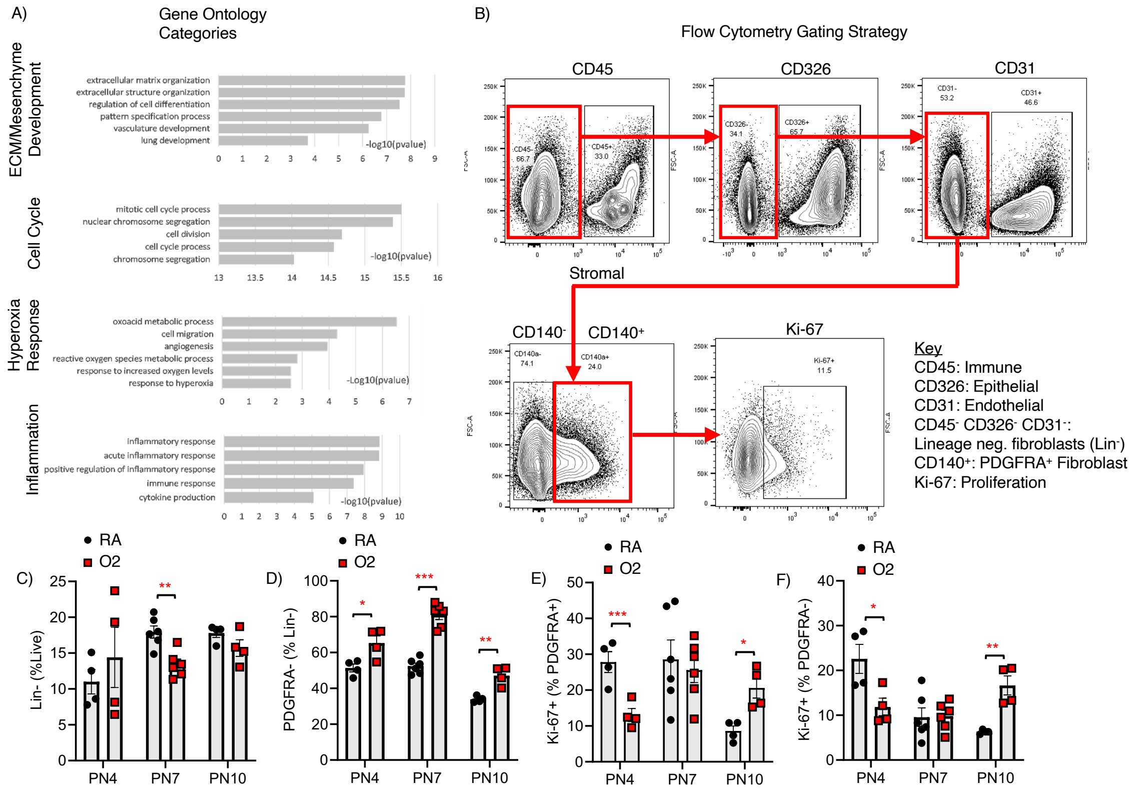

Supplemental Figure 2. Changes to PDGFRA+ fibroblast transcriptional and population signature in hyperoxia
A. Gene ontology categories enriched in Hyperoxia-exposed PDGFRA+ fibroblasts at PN4, PN7 and PN10. **B.** Flow cytometry gating strategy to determine the lineage-negative mesenchyme (Lin-) and proliferation within the PDGFRA+ and PDGFRA- fibroblasts. **C.** Percentage of total Lin- fibroblasts over total live cells. **D.** Total percentage of PDGFRA- fibroblasts in comparison to total Lin- fibroblasts. **E.** Percentage of proliferation (Ki-67+) within PDGFRA+ fibroblasts. **F.** Percentage of proliferation (Ki-67+) within PDGFRA- fibroblasts. In panels (C-F), a 2-tailed Student's t-test used, *P<0.05, **P<0.01, ***P<0.001, error bars ± SEM.

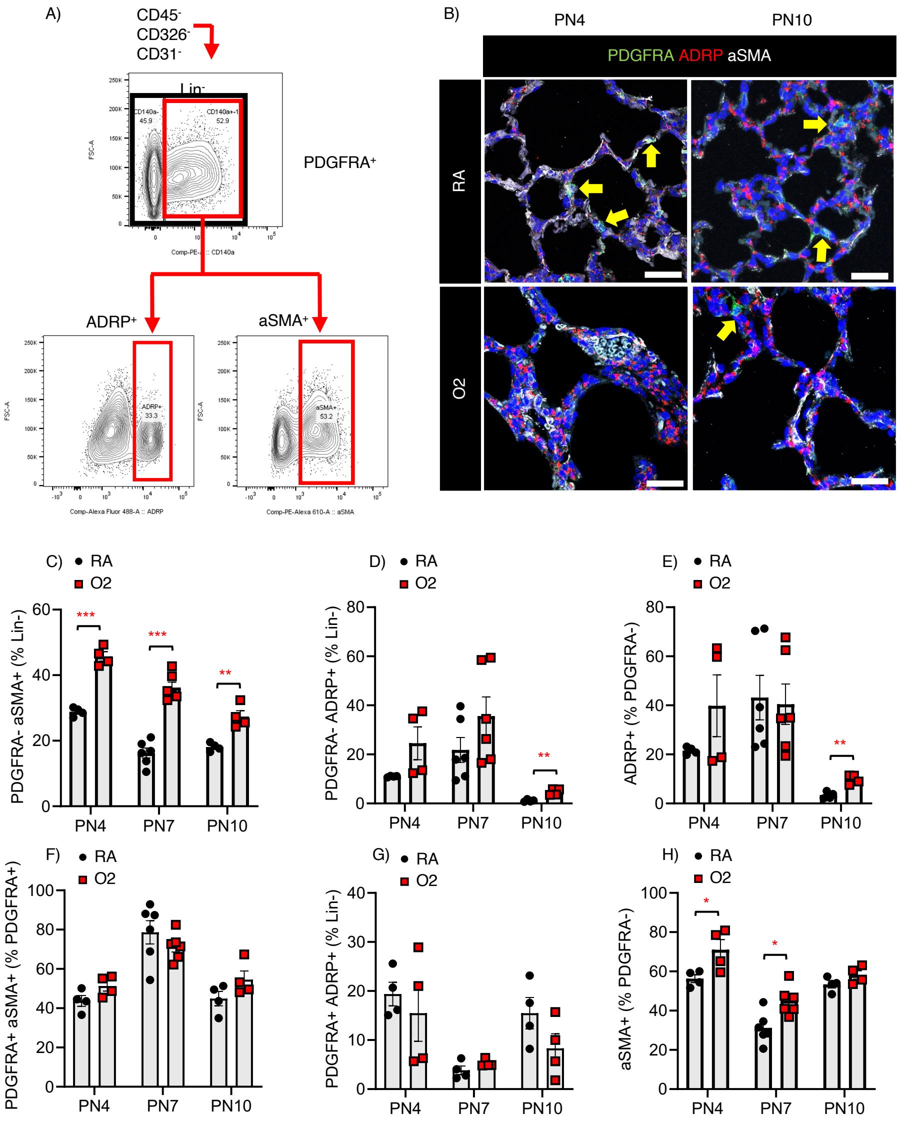

Supplemental Figure 3. Changes to lipo and myofibroblast populations in hyperoxia

A. Flow Cytometry Gating strategy on PDGFRA+ and PDGFRA- fibroblasts to determine percentage of lipofibroblasts (ADRP+) and myofibroblasts (aSMA+). **B**. Immunofluorescence of RA and O2 PN4 and PN10 lungs with PDGFRA, ADRP, and aSMA. Yellow arrows point to PDGFRA+ fibroblasts. Scale bars=25 µm **C**. Percentage of PDGFRA- aSMA+ fibroblasts compared to total Lin- fibroblasts. **D**. Percentage of PDGFRA- ADRP+ fibroblasts compared to total Lin- fibroblasts. **E.** Percentage ADRP+ fibroblasts compared to total PDGFRA- fibroblasts. **F**. Percentage of aSMA+ PDGFRA+ fibroblasts within the PDGFRA+ fibroblast population. **G**. Percentage of ADRP+ PDGFRA+ fibroblasts within the total Lin- fibroblasts. **H**. Percentage of aSMA+ fibroblasts within the PDGFRA- fibroblast population. In panels (C-H), a 2-tailed Student's t-test used, *P<0.05, **P<0.01, ***P<0.001, error bars ± SEM.

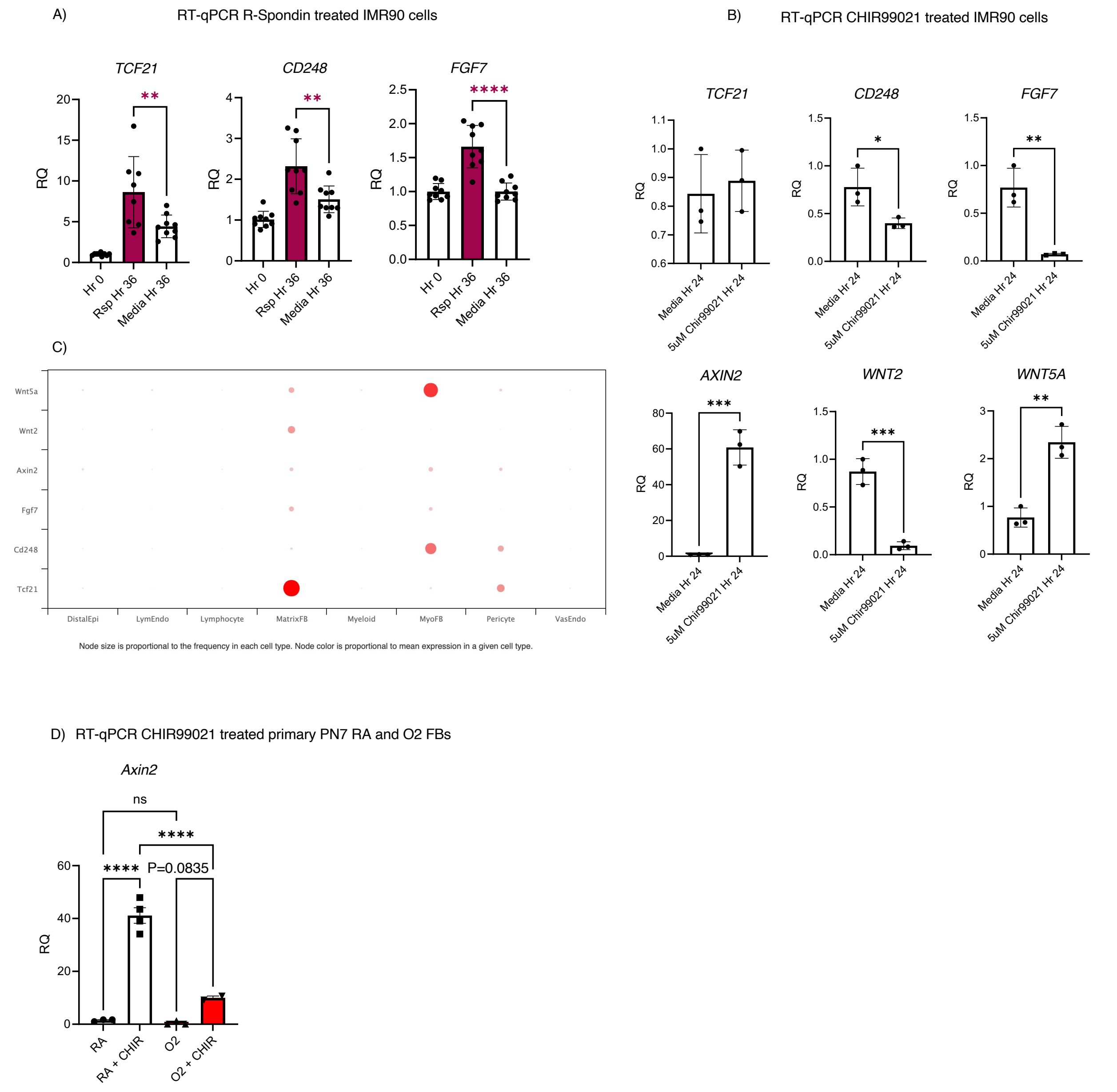

Supplemental Figure 4. Modulation of the Wnt pathway in primary and human fetal lung fibroblasts alters matrix fibroblast gene expression

A. Human PDGFRA+ embryonic lung IMR90 fibroblasts were treated in vitro for 36 hours with R-spondin conditioned media, RT-qPCR was performed for *CD248*, *FGF7*, and *TCF21*. **B**. IMR90 fibroblasts treated for 24hrs with WNT activator CHIR99021 (CHIR), RT-qPCR was performed for *CD248*, *FGF7*, *TCF21*, *AXIN2*, *WNT2*, *WNT5A*. A student's two tailed t-test was used to determine significance between two groups, *$P<0.05$, **$P<0.01$, ***$P<0.001$, error bars ± SD. **C**. Dotplot from LGEA LungGENS of PN7 signature gene expression of CD248, FGF7, TCF21, AXIN2, WNT2, WNT5A from Fluidigm C1 dataset (8). **D**. MACS-isolated PDGFRA+ FBs from RA and O2 lungs isolated and treated with or without CHIR for 24hrs. RT-qPCR performed for *Axin2*. **A, D**. A One-way ANOVA followed by Tukey's multiple comparison was used to determine significance between three groups, **$P<0.01$, ****$P<0.0001$, (A) error bars ± SD, (D) error bars ± SEM.

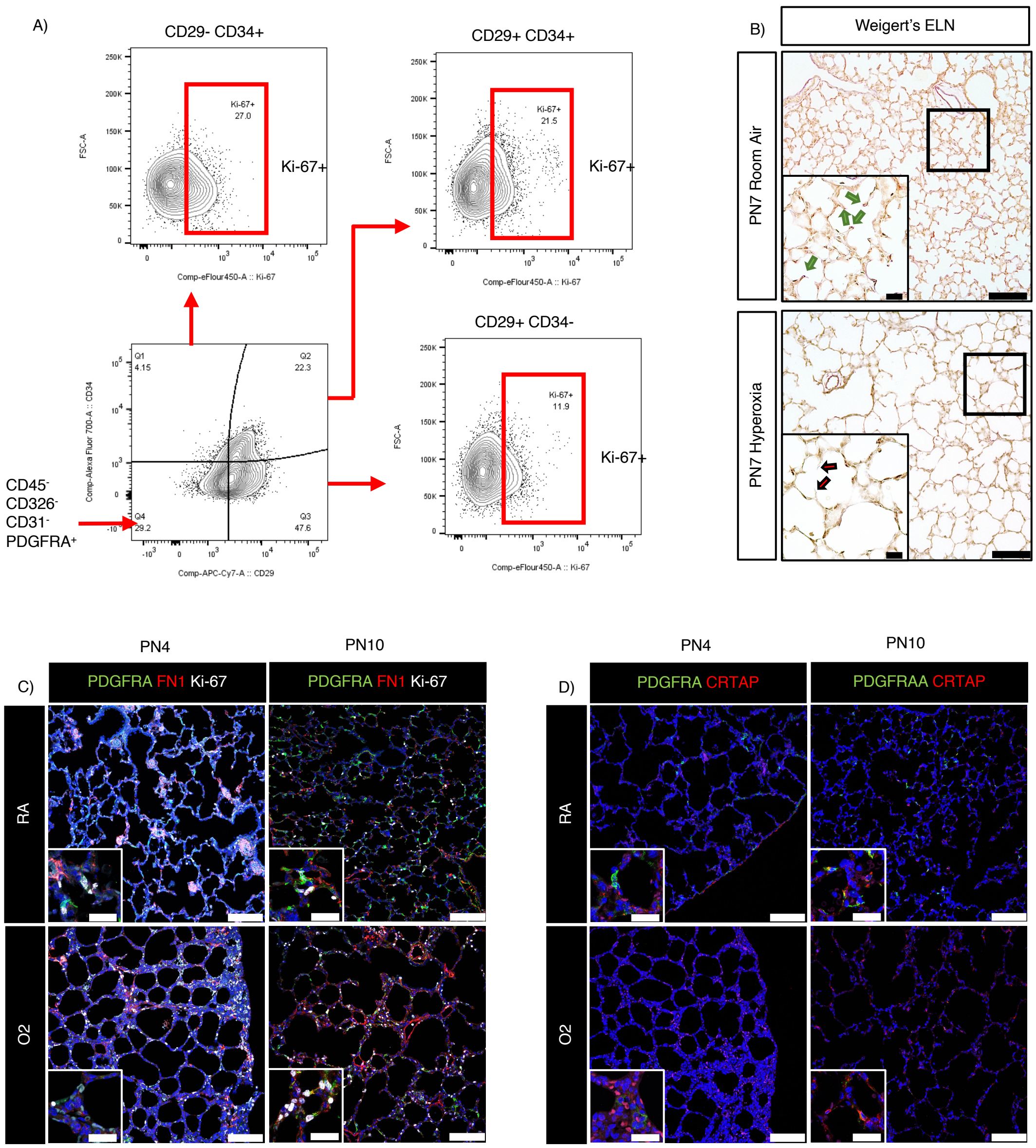

Supplemental Figure 5. PDGFRA+ Matrix fibroblast signature in hyperoxia. A. Flow cytometry gating strategy to determine proliferation (Ki-67) within PDGFRA+ CD34+ matrix fibroblasts, PDGFRA+ CD29+ myofibroblasts, and PDGFRA+ CD29+ CD34+ myo/matrix fibroblasts. **B.** Weigert's Elastin histochemical stain on PN7 room air- or hyperoxia-exposed mouse lungs. Green arrows point to alveolar entry rings with elastin, red arrows point to secondary crests that are missing elastin. Scale bars=100 µm, Inserts=25 µm**. C**. PN4, PN10 RA and O2 lungs immunofluorescence for PDGFRA, FN1, and Ki67. **D.** PN4, PN10 RA and O2 lungs immunofluorescence for CRTAP and PDGFRA. In (C, D), scale bars=100 µm, inserts 25 µm.

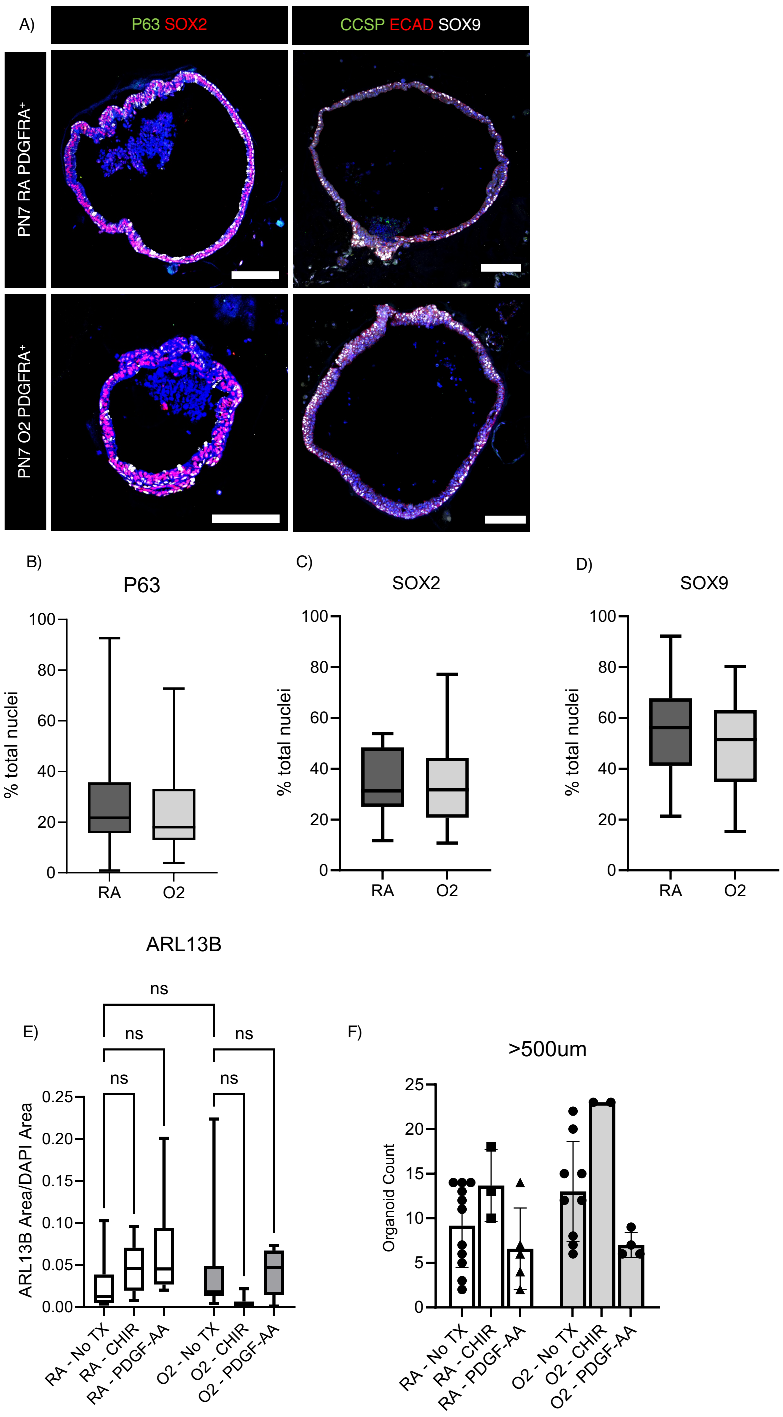

Supplemental Figure 6. Basal cells, proximal, and distal progenitors unaffected in the epithelium in organoids made with hyperoxia-exposed primary PDGFRA+ fibroblasts A. Immunofluorescence images of organoids made from PN7 room air- and hyperoxia-exposed PDGFRA+ fibroblasts and adult epithelial cells. Stained with P63, SOX2, CCSP, ECAD, and SOX9. Scale bar=100 µm **B**. Percentage of P63+ DAPI+ nuclei over total DAPI+ nuclei. **C**. Percentage of SOX2+ DAPI+ nuclei over total DAPI+ nuclei. **D**. Percentage of SOX9+ DAPI+ nuclei over total DAPI+ nuclei. In panels (B-D), a 2-tailed Student's t test was used. **E**. ARL13B immunofluorescence in organoids quantified using Nikon elements as antibody area over DAPI area. N=3-10 organoid transwells (replicates) used, 3 slides per transwell. One-way ANOVA followed by Tukey's multiple comparison was used to determine significance. **F**. Quantification of brightfield images of organoids using Nikon Elements software by size, organoids >500um diameter. One-way ANOVA followed by Tukey's multiple comparison was used to determine significance between three or more groups.

1. Grinnell F. Fibroblasts, myofibroblasts, and wound contraction. *J Cell Biol*. Feb 1994;124(4):401-4.
2. Arora PD, Narani N, McCulloch CA. The compliance of collagen gels regulates transforming growth factor-beta induction of alpha-smooth muscle actin in fibroblasts. *Am J Pathol*. Mar 1999;154(3):871-82.
3. Fiore VF, Wong SS, Tran C, et al. alphavbeta3 Integrin drives fibroblast contraction and strain stiffening of soft provisional matrix during progressive fibrosis. *JCI Insight*. Oct 18 2018;3(20)
4. Langdon WB. Performance of genetic programming optimised Bowtie2 on genome comparison and analytic testing (GCAT) benchmarks. *BioData Min*. 2015;8(1):1.
5. Plantier L, Crestani B, Wert SE, et al. Ectopic respiratory epithelial cell differentiation in bronchiolised distal airspaces in idiopathic pulmonary fibrosis. *Thorax*. Aug 2011;66(8):651-7.
6. Trapnell C, Roberts A, Goff L, et al. Differential gene and transcript expression analysis of RNA-seq experiments with TopHat and Cufflinks. *Nat Protoc*. Mar 2012;7(3):562-78.
7. Chen J, Bardes EE, Aronow BJ, Jegga AG. ToppGene Suite for gene list enrichment analysis and candidate gene prioritization. *Nucleic Acids Res*. Jul 2009;37(Web Server issue):W305-11.
8. Du Y, Kitzmiller JA, Sridharan A, et al. Lung Gene Expression Analysis (LGEA): an integrative web portal for comprehensive gene expression data analysis in lung development. *Thorax*. Jan 09 2017;
9. Xie T, Wang Y, Deng N, et al. Single-Cell Deconvolution of Fibroblast Heterogeneity in Mouse Pulmonary Fibrosis. *Cell Rep*. Mar 27 2018;22(13):3625-3640.
10. Myslobodsky M. Ingenuity pathway analysis of clozapine-induced obesity. *Obes Facts*. 2008;1(2):93-102.
11. Gokey J, Snowball J, Green J, et al. Pretreatment of aged mice with retinoic acid restores alveolar regeneration via upregulation of reciprocal PDGFRA signaling. *bioRxiv*. 2020:2020.05.06.080994.
